# *BDNF*, *DRD4*, and *HTR2A* Gene Allele Frequency Distribution and Association with Mental Illnesses in the European Part of Russia

**DOI:** 10.3390/genes15020240

**Published:** 2024-02-14

**Authors:** Anna Morozova, Valeriya Ushakova, Olga Pavlova, Sakeena Bairamova, Nika Andryshenko, Aleksandra Ochneva, Olga Abramova, Yana Zorkina, Valery A. Spektor, Timur Gadisov, Andrey Ukhov, Eugene Zubkov, Kristina Solovieva, Polina Alexeeva, Elena Khobta, Kira Nebogina, Alexander Kozlov, Tatyana Klimenko, Olga Gurina, Svetlana Shport, George Kostuyk, Vladimir Chekhonin, Konstantin Pavlov

**Affiliations:** 1 V. Serbsky National Medical Research Centre of Psychiatry and Narcology, Kropotkinsky per. 23, 119034 Moscow, Russia; ushakovavm@yandex.ru (V.U.); uovnew@mail.ru (O.P.); bairamova23s@gmail.com (S.B.); aleksochneva@yandex.ru (A.O.); abramova1128@gmail.com (O.A.); zorkina.ya@serbsky.ru (Y.Z.); gadisov.t@serbsky.ru (T.G.); ukhov.andrey@gmail.com (A.U.); zubkov.e@serbsky.ru (E.Z.); olga672@yandex.ru (O.G.); pkanew@mail.ru (K.P.); 2 Mental-Health Clinic No. 1 Named after N.A. Alekseev, Zagorodnoe Highway 2, 115191 Moscow, Russia; 3 Department of Neurobiology, M.V. Lomonosov Moscow State University, 119991 Moscow, Russia; 4 Department of Biology, MSU-BIT Shenzhen University, Shenzhen 518172, China; nika.va2001@gmail.com; 5 Department of Medical Nanobiotechnologies, Pirogov Russian National Research Medical University, 117997 Moscow, Russia

**Keywords:** mood disorders, schizophrenia spectrum disorders, mental disorders, single nucleotide polymorphism, *BDNF*, *HTR2A*, *DRD4*

## Abstract

The prevalence of mental disorders and how they are diagnosed represent some of the major problems in psychiatry. Modern genetic tools offer the potential to reduce the complications concerning diagnosis. However, the vast genetic diversity in the world population requires a closer investigation of any selected populations. In the current research, four polymorphisms, namely rs6265 in *BDNF*, rs10835210 in *BDNF*, rs6313 in *HTR2A*, and rs1800955 in *DRD4*, were analyzed in a case–control study of 2393 individuals (1639 patients with mental disorders (F20-F29, F30-F48) and 754 controls) from the European part of Russia using the TaqMan SNP genotyping method. Significant associations between rs6265 *BDNF* and rs1800955 *DRD4* and mental impairments were detected when comparing the general group of patients with mental disorders (without separation into diagnoses) to the control group. Associations of rs6265 in *BDNF*, rs1800955 in *DRD4*, and rs6313 in *HTR2A* with schizophrenia in patients from the schizophrenia group separately compared to the control group were also found. The obtained results can extend the concept of a genetic basis for mental disorders in the Russian population and provide a basis for the future improvement in psychiatric diagnostics.

## 1. Introduction

Mental disorders represent one of the major socio-economic burdens affecting developed countries. According to modern research, approximately every eighth person in the world suffers from a mental disorder [1]. The economic impact of these disorders is projected to increase over the next few decades, exceeding the current costs of treatment for cardiac disorders and cancer combined. Mental illnesses significantly reduce the quality of life of a patient and often lead to disability. One of the most debilitating among the psychiatric illnesses is schizophrenia [1,2]. Despite its low lifetime prevalence (0.8–1%), schizophrenia requires significant input from healthcare facilities.

Mood disorders represent another group of mental pathologies, which are characterized by a high incidence and pronounced negative impact on the quality of life of patients. Mood disorders include depressive-anxiety states and bipolar disorders. Anxiety disorders are the most common mental illnesses. Depression is also a highly common disorder: 5% of the world’s population suffers from depression [3]. Anxiety-depressive disorders affect all aspects of a person’s life: their productivity at work and, during their studies and their social contacts, and they also closely correlate with somatic diseases [3].

Twin and family studies confirm the close association of heritability with mental illnesses. GWAS tools have helped identify a large number of single nucleotide polymorphisms (SNPs) that are associated with various mental diseases. However, in order to achieve a sufficient level of significance, such studies must contain a large number of patients, around 100,000, which means that these studies include people from different regions, continents, and countries, where the prevalence of genetic variations may vary. Therefore, when studying separate populations where allele prevalence is approximately similar, there is an opportunity to focus on the search for disease associations with an individual SNP. Given the large number of human phenotypes and the limited number of genes, it is not surprising that pleiotropy is common in many disorders, including mental illnesses [4], especially since the symptoms of mental illnesses can be shared across a variety of diseases. An association analysis of individual SNPs in a general population of psychiatric patients can help uncover some of the targets involved in the pathogenesis of psychiatric disorders, with signs characteristic of different mental illnesses. Associations of genetic variants with disease traits, without regard to patient diagnosis, may allow one to trace this relationship.

Scientific evidence collected recently suggests a parallel influence of genetic variants on the symptoms of different mental disorders [5,6], which is currently a very interesting area of research since the identification of genetic variants associated with symptoms of psychopathologies may hold prognostic value. Therefore, one of the aims of our study was to attempt to uncover the relationship between the genetic variants under investigation and the presence of certain symptoms in patients.

Most genetic studies of mental disorders have been conducted on samples consisting of Caucasians [7,8]. Relatively few researchers have looked into other populations [9,10]. This significant blind spot has greatly limited the search for biological clues to mental illness pathogenesis in a particular population group: as a result, associations that may be detected in one population group may not exist in others [9,10,11]. For example, it was demonstrated that severe SNPs associated with major depression are more common in Chinese people than in Caucasians (Sirtuin 1 gene (*SIRT 1*) rs12415800: 45% vs. 2%; phospholysine phosphohistidine inorganic pyrophosphate phosphatase gene (*LHPP*) rs35936514: 28% vs. 6%) [12]. Thus, the same gene can be distributed differently depending on the population and group of countries. Despite the fact that the gene releases the same substances and has the same function, the frequency of the allele in the populations of different countries can vary significantly. Thus, the contribution of one gene can be unequal.

Even if alleles have a similar frequency across various populations, the effects of their alleles on the risk of developing the disease might remain specific to particular populations due to the local contributions from clinical heterogeneity, gene–environment (GxE), or gene–gene (GxG) interactions. In addition, it has also been suggested that membership in a particular geographical region can also affect the prevalence, symptomatology, etiology, outcome, and course of the illness [2,13]. Understanding the genetic architecture of mental pathologies across various populations provides valuable insight into whether population differences have a bearing on the etiologic heterogeneity of the illness.

Various genes are associated with the development of mental disorders. Among them, the *BDNF* gene is one of the best known. The *BDNF* gene encodes the brain-derived neurotrophic factor, a protein belonging to the family of protein growth factors for neurons and nerve fibers. The neurotrophin is responsible for the growth and survival of nerve cells during their development. It also stimulates and controls neurogenesis (the formation of new nerve cells) and participates in neuroplasticity. Changes in adult neurogenesis and spine density can affect learning and several forms of memory and may contribute to depression-like behavior. In addition, abnormalities in the *BDNF* gene may influence the onset of affective psychiatric disorders and schizophrenia [14]. Some studies have emphasized the association of BDNF with the development of eating disorders [15]. Changes in neurotrophin genes appear to be the most promising therapeutic targets for future research.

The rs6265 C>T mutation, also called Val66Met or G196A, is a single SNP in the *BDNF* gene, which encodes the brain-derived neurotrophic factor. In particular, this mutation is a single nucleotide substitution of “C” for “T” in chromosome 11” rs6265 C>T [16]. *BDNF* rs6265 is the most studied genetic variant because it represents a functional polymorphism where, in the presence of the mutant allele, valine is replaced by methionine (Val66Met).

GWAS studies have demonstrated there is an association between this polymorphism and risky behavior [17], as well as alcohol dependence [18]. A meta-analysis showed that carriers of the Met allele exhibit an increased risk of developing geriatric depression compared to Val/Val homozygotes [19]. No data on the frequency of the occurrence in Russia have been found to date [16].

The rs10835210 C>A mutation is a gene variation that is also located in the *BDNF* gene. It represents a single nucleotide substitution of “C” for “A” in the chromosome [20]. *BDNF* rs10835210 is a poorly studied polymorphism of the *BDNF* gene. To date, there are no data on any associations of this genetic variant with psychiatric diseases in GWAS analyses. In case–control studies, mutant allele A was associated with schizophrenia [21,22]. The involvement of the polymorphism in depression, bipolar disorder, and schizophrenia pathogenesis, as well as in response to therapy in the presence of the above-mentioned diagnoses, was shown [23]. Both the rs6265 and rs10835210 genetic variants are associated with attention deficit hyperactivity disorder, as well as with social adaptability, in different populations [24]. No data on the frequency of the occurrences in Russia have been found either [20].

The serotoninergic system plays an important role in the pathogenesis of mental disorders. In this view, the genes coding serotoninergic receptors are associated with many psychopathologies [25,26]. For example, the *HTR2A* gene coding for the serotonin receptor 2A is a therapeutic target of many antidepressants. It is well-known that serotonin constitutes one of the key neurotransmitters in the central nervous system.

The rs6313, also known as T102C, is part of a 4-SNP haplotype in the serotonin receptor 2A *HTR2A* gene responsible for serotonin reuptake, among other functions [27]. The CC genotype of *HTR2A* rs6313 has previously been found to be associated with decreased numbers of *HTR2A* receptors in the central nervous system [28]. For the *HTR2A* rs6313 genetic variant, no data on any associations with mental illness were revealed in the GWAS studies. Nevertheless, the involvement of this receptor in the development of anxiety and cognitive impairment has been shown [29]. A meta-analysis showed that the *HTR2A* rs6313 T>C polymorphism significantly correlates with a higher response rate to antidepressants in depressed patients [30]. Studies of this polymorphism relative to the response to antipsychotics have also been conducted [31,32].

Dopamine system genes are broadly associated with mental disorders. The *DRD4* gene encodes a G-protein-coupled dopamine receptor of the D4 subtype. The receptor is a member of the dopamine D2-like receptor family. This receptor is expressed in several brain regions associated with planning and rewards [33]. The dopamine D4 receptor is responsible for neuronal signaling in the mesolimbic system of the brain, which regulates emotions and complex behavior.

The rs1800955 T>C mutation in *DRD4* is located in the promoter region of the *DRD4* gene and represents a single nucleotide substitution of “T” for “C” in chromosome 11 [34]. Associations with attention deficit hyperactivity disorder, substance dependence, a number of specific personality traits, and stress response have been found [35]. The only existing GWAS study showed associations between the *DRD4* rs1800955 polymorphism and internet addiction [36]. The *DRD4* rs1800955 promoter variant is associated with traits related to impulsivity [37]. Along with that, it was demonstrated that the rs1800955 genetic variant is involved in the development of schizophrenia [38]. No data on the frequency of this SNP occurrence in Russia have been detected [34].

Polygenic risk score (PRS) prediction is a useful tool for studying the effects of genetic contribution, identifying more homogeneous phenotypes, and the stratification of patients. However, previous studies have shown that prediction accuracy decreases with increasing genetic divergence between the risk allele discovery and target datasets [39]. For example, risk prediction was only 45% as accurate in Caucasians as it was in Europeans when calculated from the GWAS of Europeans [40]. These differences can be explained by ancestry-related differences in allele frequencies, linkage disequilibrium, and other factors. Importantly, the applicability of research data from European population studies to those of non-European ancestry has not been fully assessed, leading to uncertainty as to the biological relevance of the discoveries made in European samples to non-Europeans [40].

Data considering the prevalence of genetic variants are crucial for the construction of PRS prediction models. To date, the frequency of the alleles of the selected genes in the European part of Russia has not been studied in large samples. Nevertheless, some studies dedicated to the investigation of *BDNF* rs6265, an *HTR2A* rs6313, have been performed on ethnic Russians. No association of rs6562 with schizophrenia was detected in the general sampling [41]. However, a link was detected for schizophrenia regarding early onset [42]. Along with that, it was demonstrated that the genetic variants rs6265 and rs6313 affect some clinical symptoms and suicidal behavior [43,44]. The effect of *BDNF* rs10835210 and *DRD4* rs1800955 on mental disorder development was not investigated in the ethnic Russian population.

Therefore, this study aimed to investigate the distribution of the following genetic variants: rs6265, rs10835210 in *BDNF*, rs6313 in *HTR2A*, and rs1800955 in *DRD4*, and to evaluate the genotype–phenotype relationships during severe mental disorders in a case–control study of patients with a psychiatric pathology compared to the general population in Russia. In our study, we concentrated on the European part of Russia because Russia is a multi-ethnic country with a variety of resident populations. In this view, the European part is more genetically homogenous.

## 2. Materials and Methods

### 2.1. Participants

Patients with mental disorders were recruited from Mental Health Clinic No. 1— named after N.A. Alexeev of the Department of Health of Moscow—and from the clinical departments of “V. Serbsky National Medical Research Center for Psychiatry and Narcology”. Healthy controls were recruited among volunteers who attended the periodic medical examinations. Informed consent was obtained from all participants. This study was conducted according to the guidelines of the Declaration of Helsinki. The procedures involving experiments on human subjects were performed in accordance with the ethical standards (Protocol No. 2/28.10.2020 of the Local Ethic Committee). A total of 2393 subjects took part in the study (1639 patients with mental disorders and 754 healthy volunteers). Patients with mental disorders were divided into the two groups according to the diagnosis: a mood disorders group (n = 219) and a schizophrenia spectrum disorders group (n = 1420).

The inclusion criteria included the following: patients diagnosed with schizophrenia spectrum disorders (F20-F29) and mood disorders (F30-F48) for at least a year prior during an inpatient examination; somatic well-being, implying the absence of severe and decompensated somatic and neurological diseases that may affect the diagnosis of a mental disorder; aged between 18 and 55 years; provision of written consent for the clinical interview and provision of blood samples for genetic testing. Two psychiatrists independently confirmed the diagnosis by conducting structured clinical interviews based on the ICD-10 diagnostic criteria. Therefore, the presence of the following symptoms was evaluated: hallucinations, delusions of control, delusions, catatonic symptoms, affective disorders, negative symptoms, unlawful and hetero-aggressive behavior, suicidal and auto-aggressive behavior or neurotic, psychopathic symptoms, and habit and impulse disorders [45].

The exclusion criteria for all groups included the following: the presence of concomitant mental disorders, such as an addiction to drugs and other substances, as well as to alcohol; organic mental disorders of any genesis; mental retardation; and severe somatic and chronic neurological diseases.

### 2.2. DNA Extraction and Genotyping

DNA extraction was performed from 100 µL of blood using a DNA extraction kit with the M-Sorb-Blood reagent for DNA isolation from whole blood on magnetic particles (Syntol Research and Development Company, Moscow, Russia). The extraction was performed on a KingFisher™ Flex Purification System (ThermoFisher Sci, Whalthem, MA, USA). The concentration and purification were evaluated spectrophotometrically on a Varioskan™ LUX multimode microplate reader (Thermofisher Sci). The obtained DNA samples were normalized to a concentration of 10 ng/µL and transferred on a 384 Deepwell Plate using the Robotic workstation QIAgility. The DNA samples were then added to 384-well reaction plates at a rate of 20 ng DNA per well on Freedom EVO workstations (Tecan). The plates were dried at 37 °C. The resulting Dry Banks were stored at +4 °C. TaqMan SNP genotyping (*BDNF* rs6265, *BDNF* rs10835210, *HTR2A* rs6313, *DRD4* rs1800955) was performed using Syntol genotyping kits according to the manufacturer’s recommendations on QuantStudio 5 (ThermoFisher Sci).

Allele discrimination was performed using the QuantStudio Design and Analysis Desktop Software Version 1.5.

### 2.3. Statistical Analysis

Any associations between the genetic markers and mental disorders were analyzed using the SNPStats service (www.snpstats.net (accessed on 25 July 2023)) [46]. The SNPStats service allowed us to estimate the allele frequencies, genotype frequencies, the Hardy–Weinberg equilibrium, and the association of SNPs with the response. Compliance with the Hardy–Weinberg equilibrium was estimated using the chi-square test. Statistics for genetic models were calculated by logistic regression in the SNPStats service. Data from codominant, dominant, recessive, and overdominant models were used. The dominant model assumes that the effect on penetrance manifests itself for heterozygotes and homozygotes for the predisposing allele; the recessive model assumes that the effect on penetrance manifests itself only for homozygotes; the codominant model assumes that the penetrance value of heterozygotes lies between the penetrance values for both homozygotes, but that the dependence on the risk values of homozygotes is unknown; and the overdominant model assumes that the influence on penetrance manifests itself only in heterozygotes.

Differences were considered significant at *p* < 0.05. The *p*-values for the most significant models were corrected for multiple comparisons using the FDR procedure, with the a priori threshold for statistical significance set to FDR < 0.05. The problem of multiple comparisons is a serious one in research. It consists of the fact that increasing the number of statistical hypotheses tested in the process of research leads to an increase in the probability of occurrence of errors of the first type and false conclusions about the presence of differences where they do not exist. Various statistical methods allow one to solve this problem, for example, the FDR method.

The comparison of qualitative characters was performed by χ^2^ analysis. Differences were considered significant at *p* < 0.05.

## 3. Results

Table 1 presents the quantitative characteristics of the samples studied, including healthy volunteers and patients with mental disorders.

Patients and healthy volunteers did not differ from each other by age (total medium age 44 ± 13.6) or level of education. The number of employed participants significantly differed between the groups. Thus, the number of employed healthy controls was statistically higher compared to the mood disorders group (χ^2^ = 379.1, *p* < 0.001), the schizophrenia spectrum disorder group (χ^2^ = 1594.9, *p* < 0.001), and the mental disorders group (χ^2^ = 1504.4, *p* < 0.001). Patients with mood disorders also significantly differed from the schizophrenia patients by level of employment (χ^2^ = 149.1, *p* < 0.001). Patients with mental disorders demonstrated the following distribution by disability group: I—98 (5.7%), II—988 (57.7%), III—163 (9.5%), and no disability—463 (27.0%).

In the study, 2393 samples were genotyped for four genetic markers. The distribution of the genotypes is shown in Table 2.

Significant associations were shown for several SNPs when compared between different study groups (Table 3).

During our research, we analyzed the associations of various genetic variants in the *BDNF*, *HTR2A*, and *DRD4* genes in several groups of patients. First of all, we compared the general sampling of patients with mental disorders with the control group. After that, we separately compared the schizophrenia patients to the controls and mood disorders group to the control group in the search for statistically significant associations.

We uncovered associations of three polymorphisms when comparing the general group of patients with mental disorders with the control group, but only two polymorphisms proved significant after the FDR correction. For the rs6265 genetic variant in the *BDNF* gene, significant associations were shown for homozygotes and heterozygotes compared to non-carriers in the dominant model (OR = 0.76, 95% CI = 0.62–0.92, *p* = 0.0046, FDR = 0.009); allele T was less common in the group of patients with mental disorders, suggesting a protective effect. Significant associations were also detected for homozygotes and heterozygotes compared to non-carriers in the dominant model (OR = 1.37, 95% CI = 1.13–1.66, *p* = 0.0013, FDR = 0.005) for the rs1800955 polymorphism in the *DRD4* gene; allele C was more common in the group of patients with mental disorders. The results of the genotype frequency analysis for all polymorphisms are shown in the supplementary files (Table S1).

When comparing the group of patients with mood disorders separately to the control group, associations were detected only for one genetic variant—rs1800955 (*DRD4* gene) (Table 3). Significant associations were shown for homozygotes and heterozygotes compared to non-carriers in the dominant model (OR = 1.61, 95% CI = 1.13–2.29, *p* = 0.0067, FDR = 0.03); allele C was more common in the group of patients with mood disorders. The results of the genotype frequency analysis for all polymorphisms are shown in the supplementary files (Table S2).

When comparing the control group with the group of schizophrenia spectrum disorder patients, associations were revealed for three polymorphisms. For the rs6265 polymorphism in the *BDNF* gene, significant associations were shown for homozygotes and heterozygotes compared to non-carriers in the dominant model (OR = 0.75, 95% CI = 0.61–0.91, *p* = 0.0041, FDR = 0.008); allele T was less common in the group of patients with schizophrenia spectrum disorders, suggesting a protective effect. For the rs6313 polymorphism in the *HTR2A* gene, significant associations were revealed for homozygotes and heterozygotes compared to non-carriers in the dominant model (OR = 0.81, 95% CI = 0.67–0.97, *p* = 0.02, FDR = 0.03); allele A was less common in the group of patients with schizophrenia spectrum disorders. Significant associations were also shown for homozygotes and heterozygotes compared to non-carriers in the dominant model (OR = 1.34, 95% CI = 1.10–1.63, *p* = 0.0037, FDR = 0.01) for the rs1800955 polymorphism in the *DRD4* gene (Table 3). The results of the genotype frequency analysis for all polymorphisms are presented in the supplementary files (Table S3).

We detected no statistical associations when comparing the groups of patients with schizophrenia spectrum disorders and mood disorders (schizophrenia spectrum disorder compared to mood disorders). The results of the genotype frequency analysis for all genetic variants are shown in Table 4. The results of the genotype frequency analysis for all polymorphisms are shown in the supplementary files (Table S4).

During the experiment, we also investigated significant associations with various symptoms of mental disorders for several SNPs (Table 4).

We assessed the association of genetic variants with core symptoms of schizophrenia spectrum disorders and mood disorders. Associations with symptomatology were uncovered for all four genetic variants. However, after the FDR correction, only one association was found to be significant (Table 4). For the rs1800955 polymorphism in the *DRD4* gene, significant associations were shown for heterozygotes compared to homozygotes in the overdominant model (OR = 0.75, 95% CI = 0.60–0.94, *p* = 0.011, FDR = 0.04). The results of the genotype frequency analysis for all genetic variants are shown in the supplementary files (Tables S5–S13).

## 4. Discussion

The difficulty in early diagnosing of mental disorders is one of the major problems in psychiatry. Modern genetic tools may allow one to improve the diagnosis of mental disorders by evaluating the genetic predisposition to disease development. However, the heterogeneity of various populations and environmental factors complicate the identification of potent genetic markers [47]. In this context, the evaluation of variable genetic markers in different populations is an important undertaking.

Allele frequencies vary among different populations. When comparing allele frequencies in the population of the European part of Russia, evaluated in our study, with the main European African, Asian, and Latin groups [16,20,27,34], differences were detected for all the genetic variations studied (Table 5).

The obtained differences in allele frequency for the population of the European part of Russia confirm the importance of performing genetic studies on individual populations and identifying genetic heterogeneities among different populations.

In our study, we evaluated the frequency of some gene alleles in a population of the European part of Russia and conducted a case–control study considering patients with mental disorders.

The results of this study confirmed the association of rs6265 polymorphisms in the *BDNF* gene with mental disorders. This association was significant for the general cohort of patients regardless of the diagnosis. After the division of the entire cohort of patients into subgroups by the diagnoses (mood disorders and schizophrenia), the *BDNF* polymorphism was found to be associated only with schizophrenia, whereas no links were detected for mood disorders. According to our research, the rs10835210 *BDNF* is not associated with mental disorders.

The demonstrated association of the rs6265 polymorphism is rather expected. BDNF plays a major role in the functioning of the nervous system. It is suggested that the Met allele disrupts intracellular traffic and synaptic localization for the mature BDNF and significantly attenuates active BDNF secretion (up to 30%). This polymorphism affects the anatomy of the hippocampus and prefrontal cortex in healthy individuals. Met carriers showed a significant bilateral reduction in hippocampal gray matter volume compared to Val homozygotes, suggesting a modifying effect of the functional variation on the brain morphology associated with learning and memory [48].

The involvement of BDNF in the pathogenesis of psychiatric disorders and responses to therapy has been widely demonstrated [49]. A solid body of data supports the association of rs6265 with mood disorders [50,51] and schizophrenia [52], although some studies do not [53]. The link between *BDNF* rs6265 polymorphisms and psychiatric disorders was also revealed in studies that investigated the Russian population [51,54]. In general, a number of studies performed on populations of European ancestry indicate an association between the rs6265 genetic variant and schizophrenia development and symptoms. For example, such results were obtained for the Armenian population [55], Polish population [56], and Scottish population [57]. Along with that, a positive link with the age of schizophrenia onset was indicated in African-Americans [58]. The studies that have investigated the Asian population are rather inconclusive. Some data reveal an association with schizophrenia in the Chinese Han population [59,60]. Nevertheless, a larger amount of studies have found no association between rs6265 and schizophrenia in the Asian population [61,62]. For example, such results were arrived at for the Japanese and Malay populations [63,64,65]. Thus, the demonstrated heterogeneity of phenotypes in different populations indicates how relevant the study of monoethnic samples is.

Along with the study of disease associations, some researchers have concentrated on the search for genetic variant associations with various symptoms. In particular, it has been shown that men with the val/met genotype present an earlier age of onset and that the val/val genotype predisposes them to more severe symptomatology, especially on the general psychopathology scale of the Positive and Negative Symptom Scale [56]. Depressed patients who carry the *BDNF* Val66Met polymorphism variant (GA + AA) demonstrate a significantly increased risk of suicidal behavior [66]. Associations have also been shown with symptom severity on the Hamilton scale [51] and with the risk of developing chronic depression [67]. In depressed patients, the met allele has been associated with a better response to antidepressants [68,69] and psychological therapies for depression [70].

Patients with bipolar disorders who carried the *BDNF* met allele exhibited smaller hippocampal volumes and scored lower on the cognitive scale compared to depressed patients and healthy controls [71]. Our research revealed no association of the genetic variant with pathological symptoms.

Regarding *BDNF* rs10835210, no association of the polymorphism with mental disorders has been detected in the Russian population despite the positive results obtained in studies by research groups from other countries [22,72]. It is of interest that the association of the rs10835210 genetic variant with schizophrenia was first revealed in the Han Chinese population [73]. These efforts demonstrated an association between genetic variants and symptoms of the disease [22,73]. Along with that, Zhang et al. demonstrated that the rs10835210 mutant A allele affects a person’s susceptibility to schizophrenia [22]. This may be an indirect indication that ethnic and geographical factors can influence the genetic heterogeneity of different populations.

Furthermore, rs10835210 has been associated with internalizing disorders [74], suicide attempts [75], and phobic disorders [76]. An association of life stress with depressive symptoms and non-suicidal self-harm, as well as an anxious mood with executive function and depressed mood with intentional self-harm, have also been shown in healthy individuals. Each of these BDNF × stress/mood interactions was characterized by stress/mood associations that were stronger in individuals with the AA genotype (homozygous for the minor allele) than in individuals with a genotype that included a major allele (AC or CC) [77]. Nevertheless, it is worth noting that far fewer studies considering rs10835210 have been conducted to date.

For rs6313 in the *HTR2A* gene, a significant association was found only in the schizophrenia subgroup. For the general patient population and for the mood disorders subgroup separately, the significance of this polymorphism evaporated after the FDR correction.

The involvement of 5HT2A receptors in mental disease pathogenesis has been demonstrated [78]. This is partly confirmed by the application of the antipsychotic aripiprazole, which antagonizes 5HT2A receptors, in the therapy of schizophrenia [79]. For the rs6313 polymorphism, the data in the literature demonstrate an association with the cognitive function level [80] and Alzheimer’s disease [81] and show a nominal association with panic disorder [82]. The connection of psychiatric disorders with the rs6313 polymorphism in *5HT2A* has also been investigated in the Russian population [83,84]. In particular, polymorphism has been shown to contribute to the development of suicidal behavior in individuals with addictions and schizophrenia patients [44,83]. Along with that, the association of rs6313 with symptoms of schizophrenia has also been detected in the Russian population [43].

Regarding research into other populations, a number of studies do not indicate a statistical association of *HTR2A* rs6313 with schizophrenia. In particular, such results were demonstrated for the Turkish [85], Chinese Han [86], Iranian [87], and North Indian populations [88]. However, Sujitha et al. have revealed an rs6313 association with schizophrenia in the Tamil-speaking population of South India [89]. It is interesting that Wang et al. have shown that polymorphism can affect antipsychotic drug susceptibility depending on ethnicity [90]. Taken as a compound, this data reflects the influence of environmental and geographical factors on genes and acknowledges the relevance of a study of genetic alleles’ frequency and association in various populations.

Several studies have linked the polymorphism to negative symptoms in schizophrenia [91]. Along with that, the rs6313 genetic variant has been shown to be related to an increased level of aggression, impulsivity, and suicidal ideation [92]. It was noted that the relationship with impulsivity under the influence of the 5-HT2A receptor strongly depends on the context of the situation [93]. Our research revealed no association of rs6313 with mental disorders symptoms.

*DRD4* rs1800955 was associated with mental illnesses in the general group of psychiatric disorders and in the subgroups of schizophrenia and mood disorders separately. Earlier, it was demonstrated that DRD4 is connected with mental disease pathophysiology. For example, preclinical research revealed that stimulation of the receptor may produce a positive effect on cognitive function in schizophrenia [94]. Some studies have shown an association between the rs1800955 polymorphism and autism spectrum disorders [95], smoking [96], and hyperactivity [97]. In the Russian population, polymorphism has been investigated in the field of antipsychotic drug assessment [84] and opioid dependence [98]. However, these studies have not uncovered significant associations.

Regarding schizophrenia development, no association with disease susceptibility has been demonstrated in the Japanese population [99]. Frydecka et al. also established no effect of rs1800955 on learning in schizophrenia [100]. However, the research of Tsutsumi et al. demonstrated an association of the polymorphism with the response to antipsychotic drugs [101]. The limited pool of data on *DRD4* rs10800955 effects indicates the need to further investigate the genetic variant association with mental disorders.

Along with the association with various mental disorders, our study revealed the association of rs1800955 with delusions. To date, there are no data in scientific publications on the association of the rs1800955 polymorphism in the *DRD4* gene with delusional symptoms. However, this genetic variant has been shown to be associated with another psychopathologic symptom, in particular, paranoid symptoms in patients with methamphetamine use disorder [102]. At the same time, there are data indicating that other *DRD4* gene variants are associated with delusions. For example, our results can be compared with the results obtained by Serretti et al., who studied *DRD4* in different psychiatric disorders (bipolar disorder, depression, schizophrenia, and delusional disorder) on a large sample of 2011 patients. That analysis suggests that the *DRD4* genetic variant exerts a small but significant influence on delusional symptoms in patients affected by major psychoses [103]. Another study performed by the same authors showed that functional variants in the *DRD4* gene may be associated with delusional symptoms in mood disorders [104]. Catalano et al. have shown that genetic variations (polymorphic 12 base pair repeat) in the *DRD4* gene lead to a predisposition to delusional disorder [105]. These data suggest that *DRD4* genetic variants may affect the risks of delusional symptom development in patients with mental disorders, a point that was confirmed by the results of our study.

Our study also demonstrated that the schizophrenia and mood disorder subgroups did not differ based on the genetic associations studied. This result confirms the findings of a common genetic basis for many psychiatric disorders and suggests a substantial pleiotropy of contributing loci. Indeed, some studies suggest a shared common genetic pattern in these disorders [106,107]. However, the associations found for the schizophrenia group compared to the controls may be indirect evidence of a stronger genetic basis for this disorder compared to mood disorders.

Our study has some limitations. The main limitation is the rather small sample of mood disorder patients. Nevertheless, the absence of differences between the schizophrenia subgroup and mood disorders subgroup may be evidence of compatible genetic patterns for these disorders. Another limitation of our research is the absence of a cognitive decline evaluation. Given that it represents an important task requiring serious research, in our manuscript, we decided to concentrate primarily on mental and mood symptoms.

## 5. Conclusions

This study has uncovered significant associations between rs6265 in *BDNF*, rs1800955 in *DRD4*, and psychiatric disorders in a general cohort of patients with mental illnesses compared to the controls. The research also established a link between rs6265 in *BDNF*, rs1800955 in *DRD4*, and rs6313 in *HTR2A* and schizophrenia in patients with schizophrenia spectrum disorder compared to the control group. Along with that, an association of rs1800955 in *DRD4* with mood disorders and delusions was also demonstrated. The obtained results have the potential to extend the concept of a genetic basis of mental disorders in the Russian population and contribute additional data towards the future improvement in psychiatric diagnostics.

## Figures and Tables

**Table 1:** Quantitative characteristics of healthy volunteers and patients.

	All Subjects	Control	Mental Disorders	Schizophrenia Spectrum Disorders	Mood Disorders
Number of participants	2393	754	1639	1420	219
Sex, N	1126 males 1267 females	296 males 458 females	830 males 809 females	765 males 655 females	65 males 154 females
Age, Mean (SD)	44 (13.6)	42 (15.2)	46 (13.2)	46 (12.9)	44 (14.9)
Higher Education (%)	776 (32.4%)	263 (34.9%)	513 (31.3%)	441 (31.1%)	72 (32.9%)
Employment (%)	906 (37.9%)	713 (94.5%)	193 (11.8%)	80 (36.5%)	113 (8%)

**Table 2:** Frequency of alleles and testing for the Hardy–Weinberg equilibrium.

SNP	All Subjects	Control	Mental Disorders	Mood Disorders	Schizophrenia Spectrum Disorders	Test for the Hardy–Weinberg Equilibrium (*p*-Value) *
*BDNF rs6265 (C>T)*						
T/T	54 (2%)	22 (3%)	32 (2%)	6 (3%)	26 (2%)	0.51
C/T	580 (25%)	207 (28%)	373 (23%)	51 (24%)	322 (23%)	
C/C	1732 (73%)	519 (69%)	1213 (75%)	159 (73%)	1054 (75%)	
	T = 0.15	T = 0.17	T = 0.14	T = 0.15	T = 0.13	
*BDNF rs10835210 (C>A)*						
C/C	588 (25%)	197 (28%)	391 (24%)	67 (31%)	342 (25%)	0.41
A/A	588 (25%)	170 (24%)	418 (26%)	49 (23%)	351 (25%)	
C/A	1135 (50%)	346 (48%)	789 (50%)	101 (46%)	688 (50%)	
	A = 0.5	A = 0.48	A = 0.51	A = 0.46	A = 0.5	
*HTR2A rs6313 (G>A)*						
A/A	294 (13%)	111 (15%)	183 (12%)	21 (10%)	162 (12%)	0.52
G/G	941 (42%)	289 (39%)	652 (43%)	78 (38%)	574 (44%)	
G/A	1022 (45%)	345 (46%)	677 (45%)	105 (52%)	572 (44%)	
	A = 0.36	A = 0.38	A = 0.34	A = 0.36	A = 0.34	
*DRD4 rs1800955 (T>C)*						
C/C	497 (22%)	133 (19%)	364 (23%)	44 (21%)	320 (23%)	0.38
T/T	670 (29%)	238 (34%)	432 (27%)	51 (24%)	381 (28%)	
T/C	1111 (49%)	328 (47%)	783 (50%)	115 (55%)	668 (49%)	
	C = 0.46	C = 0.42	C = 0.48	C = 0.48	C = 0.48	

* The test for the Hardy–Weinberg equilibrium was performed for the total cohort.

**Table 3:** Analysis of allele frequency association with study groups.

Genes	SNP	Association of Allele Frequency with Study Groups		
		The Most Significant Model	*p*-Value	FDR *
Mental disorders compared to controls				
*BDNF*	rs6265	Dominant	**0.0046**	**0.009**
*BDNF*	rs10835210	Recessive	0.11	0.11
*HTR2A*	rs6313	Dominant	**0.049**	0.06
*DRD4*	rs1800955	Dominant	**0.0013**	**0.005**
Mood disorders compared to controls				
*BDNF*	rs6265	Dominant and Overdominant	0.23	0.23
*BDNF*	rs10835210	Recessive	**0.04**	0.08
*HTR2A*	rs6313	Recessive	0.083	0.11
*DRD4*	rs1800955	Dominant	**0.0067**	**0.03**
Schizophrenia spectrum disorders compared to controls				
*BDNF*	rs6265	Dominant	**0.0041**	**0.008**
*BDNF*	rs10835210	Dominant	0.16	0.16
*HTR2A*	rs6313	Dominant	**0.02**	**0.03**
*DRD4*	rs1800955	Dominant	**0.0037**	**0.01**
Mood disorders compared to schizophrenia spectrum disorders				
*BDNF*	rs6265	Recessive	0.39	0.39
*BDNF*	rs10835210	Dominant	0.094	0.19
*HTR2A*	rs6313	Overdominant	**0.04**	0.16
*DRD4*	rs1800955	Overdominant	0.11	0.15

* FDR: false discovery rate; *p*-value < 0.05 was considered significant (marked in bold).

**Table 4:** Analysis of allele frequency association with symptom clusters (symptoms observed/no symptoms observed).

Genes	SNP	Association of Allele Frequency with Study Groups		
		The Most Significant Model	*p*-Value	FDR *
Hallucinations				
*BDNF*	rs6265	Overdominant	0.24	0.32
*BDNF*	rs10835210	Recessive	0.12	0.24
*HTR2A*	rs6313	Overdominant	0.55	0.55
*DRD4*	rs1800955	Recessive	**0.025**	0.1
Delusions of control				
*BDNF*	rs6265	Overdominant	0.29	0.29
*BDNF*	rs10835210	Dominant	0.07	0.09
*HTR2A*	rs6313	Recessive	**0.033**	0.07
*DRD4*	rs1800955	Dominant	**0.022**	0.09
Delusions				
*BDNF*	rs6265	Recessive	0.59	0.59
*BDNF*	rs10835210	Dominant	0.45	0.60
*HTR2A*	rs6313	Overdominant	0.23	0.46
*DRD4*	rs1800955	Overdominant	**0.011**	**0.04**
Catatonic symptoms				
*BDNF*	rs6265	Overdominant	0.17	0.34
*BDNF*	rs10835210	Recessive	0.3	0.30
*HTR2A*	rs6313	Recessive	0.23	0.31
*DRD4*	rs1800955	Recessive	0.06	0.24
Neurotic, psychopathic symptoms, and habit and impulse disorders				
*BDNF*	rs6265	Recessive	0.48	0.64
*BDNF*	rs10835210	Dominant	**0.016**	0.06
*HTR2A*	rs6313	Overdominant	0.32	0.64
*DRD4*	rs1800955	Overdominant	0.73	0.73
Suicidal and auto-aggressive behavior				
*BDNF*	rs6265	Recessive	**0.013**	0.052
*BDNF*	rs10835210	Recessive	0.73	0.73
*HTR2A*	rs6313	Recessive	0.35	0.47
*DRD4*	rs1800955	Dominant	0.15	0.30
Unlawful and hetero-aggressive behavior				
*BDNF*	rs6265	Recessive	0.18	0.24
*BDNF*	rs10835210	Overdominant	0.4	0.40
*HTR2A*	rs6313	Overdominant	**0.026**	0.052
*DRD4*	rs1800955	Dominant	**0.025**	0.10
Affective disorders				
*BDNF*	rs6265	Dominant	0.07	0.28
*BDNF*	rs10835210	Dominant	0.17	0.34
*HTR2A*	rs6313	Recessive	0.22	0.22
*DRD4*	rs1800955	Overdominant	0.17	0.23
Negative symptoms				
*BDNF*	rs6265	Dominant	0.61	0.61
*BDNF*	rs10835210	Overdominant	0.09	0.36
*HTR2A*	rs6313	Overdominant	0.18	0.36
*DRD4*	rs1800955	Recessive	0.25	0.33

* FDR: false discovery rate; *p*-value < 0.05 was considered significant (marked in bold).

**Table 5:** Comparison of the allele frequencies mentioned in our study in the control group with allele frequencies in different populations (European, African, Asian, and Latin groups).

	European	African	Asian	Latin
*BDNF* rs6268	T = 0.193746 χ^2^ = 3.795 *p* = 0.052	T = 0.04243 χ^2^ = 154.6 ***p* < 0.001**	T = 0.4449 χ^2^ = 360 ***p* < 0.001**	A = 0.15765 χ^2^ = 0.667 *p* = 0.415
*BDNF* rs10835210	A = 0.433849 χ^2^ = 6.371 ***p* = 0.012**	A = 0.0823 χ^2^ = 1523 ***p* < 0.001**	A = 0.163 χ^2^ = 84.816 ***p* < 0.001**	A = 0.23 χ^2^ = 268 ***p* < 0.001**
*HTR2A* rs6313	A = 0.419754 χ^2^ = 5.388 ***p* = 0.021**	A = 0.37809 χ^2^ = 0.023 *p* = 0.879	A = 0.5515 χ^2^ = 127 ***p* < 0.001**	A = 0.387 χ^2^ = 0.151 *p* = 0.698
*DRD4* rs1800955	C = 0.44079 χ^2^ = 0.967 *p* = 0.326	C = 0.3844 χ^2^ = 6.272 ***p* = 0.013**	C = 0.295 χ^2^ = 7.245 ***p* = 0.008**	C = 0.382 χ^2^ = 3.231 *p* = 0.073
		A *p*-value < 0.05 was considered significant (marked in bold).		

## Data Availability

Data are available on request due to restrictions on privacy.

## Supplementary Materials

The following supporting information can be downloaded at: https://www.mdpi.com/article/10.3390/genes15020240/s1, Table S1: analysis of the association of allele frequency with the study groups (mental disorders to controls); Table S2: analysis of the association of allele frequency with the study groups (control to mood disorders); Table S3: analysis of the association of allele frequency with the study groups (control to schizophrenia spectrum disorders); Table S4: analysis of the association of allele frequency with the study groups (mood disorders to schizophrenia spectrum disorders); Table S5: analysis of the association of allele frequency with the symptom cluster—hallucinations (symptoms observed/no symptoms observed); Table S6: analysis of the association of allele frequency with the symptom cluster—delusions of control (symptoms observed/no symptoms observed); Table S7: analysis of the association of allele frequency with symptom cluster—delusions (symptoms observed/no symptoms observed); Table S8: analysis of the association of allele frequency with symptom cluster—catatonic symptoms (symptoms observed/no symptoms observed); Table S9: analysis of the association of allele frequency with symptom cluster—neurotic, psychopathic symptoms, and habit and impulse disorders (symptoms observed/no symptoms observed); Table S10: analysis of the association of allele frequency with the symptom cluster—suicidal and auto-aggressive behavior (symptoms observed/no symptoms observed); Table S11: analysis of the association of allele frequency with the symptom cluster—unlawful and hetero-aggressive behavior (symptoms observed/no symptoms observed), Table S12: analysis of the association of allele frequency with symptom cluster—affective disorders (symptoms observed/no symptoms observed); Table S13: analysis of the association of allele frequency with symptom cluster—negative symptoms (symptoms observed/no symptoms observed).
