# Supplementary material for: BDNF, DRD4, and HTR2A Gene Allele Frequency Distribution and Association with Mental Illnesses in the European Part of Russia
Source: Genes (Basel). 2024 Feb 14;15(2):240. doi: 10.3390/genes15020240 (PMC10887670; doi:10.3390/genes15020240)
Supplement: Supplementary file 1 [file genes-15-00240-s001.zip › genes-2775310-supplementary.pdf]

Supplementary materials

Table S1: Analysis of the association of allele frequency with the study groups (**mental disorders to controls**).

**BDNF rs6265 association with response Group (n=2366, crude analysis)**							
**Model**	**Genotype**	**Control**	**Mental Disorders**	**OR (95% CI)**	**P-value**	**AIC**	**BIC**
Codominant	C/C	519 (69.4%)	1213 (75%)	1.00	0.014	2949.9	2967.2
	C/T	207 (27.7%)	373 (23.1%)	**0.77 (0.63-0.94)**			
	T/T	22 (2.9%)	32 (2%)	0.62 (0.36-1.08)			
Dominant	C/C	519 (69.4%)	1213 (75%)	1.00	0.0046	2948.4	2960
	C/T–T/T	229 (30.6%)	405 (25%)	**0.76 (0.62-0.92)**			
Recessive	C/C–C/T	726 (97.1%)	1586 (98%)	1.00	0.15	2954.4	2965.9
	T/T	22 (2.9%)	32 (2%)	0.67 (0.38-1.15)			
Overdominant	C/C-T/T	541 (72.3%)	1245 (77%)	1.00	0.016	2950.6	2962.2
	C/T	207 (27.7%)	373 (23.1%)	**0.78 (0.64-0.95)**			
**BDNF rs10835210 association with response Group (n=2311, crude analysis)**							
**Model**	**Genotype**	**Control**	**Mental Disorders**	**OR (95% CI)**	**P-value**	**AIC**	**BIC**
Codominant	A/A	170 (23.8%)	418 (26.2%)	1.00	0.22	2859	2876.2
	C/A	346 (48.5%)	789 (49.4%)	0.93 (0.75-1.15)			
	C/C	197 (27.6%)	391 (24.5%)	0.81 (0.63-1.03)			
Dominant	A/A	170 (23.8%)	418 (26.2%)	1.00	0.24	2858.6	2870.1
	C/A-C/C	543 (76.2%)	1180 (73.8%)	0.88 (0.72-1.09)			
Recessive	A/A-C/A	516 (72.4%)	1207 (75.5%)	1.00	0.11	2857.4	2868.9
	C/C	197 (27.6%)	391 (24.5%)	0.85 (0.69-1.04)			
Overdominant	A/A-C/C	367 (51.5%)	809 (50.6%)	1.00	0.71	2859.9	2871.4
	C/A	346 (48.5%)	789 (49.4%)	1.03 (0.87-1.23)			
**HTR2A rs6313 association with response Group (n=2257, crude analysis)**							
**Model**	**Genotype**	**Control**	**Mental Disorders**	**OR (95% CI)**	**P-value**	**AIC**	**BIC**
Codominant	G/G	289 (38.8%)	652 (43.1%)	1.00	0.065	2863.5	2880.7
	G/A	345 (46.3%)	677 (44.8%)	0.87 (0.72-1.05)			
	A/A	111 (14.9%)	183 (12.1%)	**0.73 (0.56-0.96)**			
Dominant	G/G	289 (38.8%)	652 (43.1%)	1.00	0.049	2863.1	2874.5
	G/A-A/A	456 (61.2%)	860 (56.9%)	0.84 (0.70-1.00)			
Recessive	G/G-G/A	634 (85.1%)	1329 (87.9%)	1.00	0.066	2863.6	2875
	A/A	111 (14.9%)	183 (12.1%)	0.79 (0.61-1.01)			
Overdominant	G/G-A/A	400 (53.7%)	835 (55.2%)	1.00	0.49	2866.5	2877.9
	G/A	345 (46.3%)	677 (44.8%)	0.94 (0.79-1.12)			
**DRD4 rs1800955 association with response Group (n=2278, crude analysis)**							
**Model**	**Genotype**	**Control**	**Mental Disorders**	**OR (95% CI)**	**P-value**	**AIC**	**BIC**
Codominant	T/T	238 (34%)	432 (27.4%)	1.00	0.0031	2803.4	2820.6
	T/C	328 (46.9%)	783 (49.6%)	**1.32 (1.07-1.61)**			
	C/C	133 (19%)	364 (23.1%)	**1.51 (1.17-1.94)**			
Dominant	T/T	238 (34%)	432 (27.4%)	1.00	0.0013	2802.7	2814.2
	T/C-C/C	461 (66%)	1147 (72.6%)	**1.37 (1.13-1.66)**			
Recessive	T/T-T/C	566 (81%)	1215 (77%)	1.00	0.03	2808.3	2819.8
	C/C	133 (19%)	364 (23.1%)	**1.27 (1.02-1.59)**			
Overdominant	T/T-C/C	371 (53.1%)	796 (50.4%)	1.00	0.24	2811.6	2823.1
	T/C	328 (46.9%)	783 (49.6%)	1.11 (0.93-1.33)			

AIC - Akaike information criterion; BIC - Bayesian information criterion; OR - Odds ratio; P-value < 0.05 was considered as significance;

Table S2: Analysis of the association of allele frequency with the study groups (Control to Mood disorders).

**BDNFrs6265 association with response Diagnosis (n=964, crude analysis)**							
**Model**	**Genotype**	**Control**	**Mood Disorders**	**OR (95% CI)**	**P-value**	**AIC**	**BIC**
Codominant	C/C	519 (69.4%)	159 (73.6%)	1.00	0.47	1030.2	1044.8
	C/T	207 (27.7%)	51 (23.6%)	0.80 (0.56-1.15)			
	T/T	22 (2.9%)	6 (2.8%)	0.89 (0.35-2.23)			
Dominant	C/C	519 (69.4%)	159 (73.6%)	1.00	0.23	1028.3	1038
	C/T–T/T	229 (30.6%)	57 (26.4%)	0.81 (0.58-1.14)			
Recessive	C/C–C/T	726 (97.1%)	210 (97.2%)	1.00	0.9	1029.7	1039.4
	T/T	22 (2.9%)	6 (2.8%)	0.94 (0.38-2.36)			
Overdominant	C/C-T/T	541 (72.3%)	165 (76.4%)	1.00	0.23	1028.3	1038
	C/T	207 (27.7%)	51 (23.6%)	0.81 (0.57-1.15)			
**BDNF rs10835210 association with response Diagnosis (n=930, crude analysis)**							
**Model**	**Genotype**	**Control**	**Mood Disorders**	**OR (95% CI)**	**P-value**	**AIC**	**BIC**
Codominant	C/C	197 (27.6%)	49 (22.6%)	1.00	0.087	1011.6	1026.1
	C/A	346 (48.5%)	101 (46.5%)	1.17 (0.80-1.72)			
	A/A	170 (23.8%)	67 (30.9%)	**1.58 (1.04-2.42)**			
Dominant	C/C	197 (27.6%)	49 (22.6%)	1.00	0.14	1012.3	1021.9
	C/A-A/A	516 (72.4%)	168 (77.4%)	1.31 (0.92-1.87)			
Recessive	C/C-C/A	543 (76.2%)	150 (69.1%)	1.00	0.04	1010.3	1019.9
	A/A	170 (23.8%)	67 (30.9%)	**1.43 (1.02-2.00)**			
Overdominant	C/C-A/A	367 (51.5%)	116 (53.5%)	1.00	0.61	1014.2	1023.9
	C/A	346 (48.5%)	101 (46.5%)	0.92 (0.68-1.25)			
**HTR2A rs6313 association with response Diagnosis (n=949, crude analysis)**							
**Model**	**Genotype**	**Control**	**Mood Disorders**	**OR (95% CI)**	**P-value**	**AIC**	**BIC**
Codominant	G/G	289 (38.8%)	78 (38.2%)	1.00	0.17	990.3	1004.9
	G/A	345 (46.3%)	105 (51.5%)	1.13 (0.81-1.57)			
	A/A	111 (14.9%)	21 (10.3%)	0.70 (0.41-1.19)			
Dominant	G/G	289 (38.8%)	78 (38.2%)	1.00	0.88	991.8	1001.5
	G/A-A/A	456 (61.2%)	126 (61.8%)	1.02 (0.74-1.41)			
Recessive	G/G-G/A	634 (85.1%)	183 (89.7%)	1.00	0.083	988.8	998.5
	A/A	111 (14.9%)	21 (10.3%)	0.66 (0.40-1.07)			
Overdominant	G/G-A/A	400 (53.7%)	99 (48.5%)	1.00	0.19	990.1	999.8
	G/A	345 (46.3%)	105 (51.5%)	1.23 (0.90-1.68)			
**DRD4 rs1800955 association with response Diagnosis (n=909, crude analysis)**							
**Model**	**Genotype**	**Control**	**Mood Disorders**	**OR (95% CI)**	**P-value**	**AIC**	**BIC**
Codominant	T/T	238 (34%)	51 (24.3%)	1.00	0.024	981.2	995.7
	T/C	328 (46.9%)	115 (54.8%)	**1.64 (1.13-2.37)**			
	C/C	133 (19%)	44 (20.9%)	1.54 (0.98-2.44)			
Dominant	T/T	238 (34%)	51 (24.3%)	1.00	0.0067	979.3	988.9
	T/C-C/C	461 (66%)	159 (75.7%)	**1.61 (1.13-2.29)**			
Recessive	T/T-T/C	566 (81%)	166 (79%)	1.00	0.54	986.3	995.9
	C/C	133 (19%)	44 (20.9%)	1.13 (0.77-1.65)			
Overdominant	T/T-C/C	371 (53.1%)	95 (45.2%)	1.00	0.046	982.7	992.3
	T/C	328 (46.9%)	115 (54.8%)	1.37 (1.00-1.87)			

AIC - Akaike information criterion; BIC - Bayesian information criterion; OR - Odds ratio; P-value < 0.05 was considered as significance;

Table S3: Analysis of the association of allele frequency with the study groups (Control **to** Schizophrenia spectrum disorders).

**BDNF rs6265 association with response Diagnosis (n=2150, crude analysis)**							
**Model**	**Genotype**	**Control**	**Schizophrenia spectrum disorders**	**OR (95% CI)**	**P-value**	**AIC**	**BIC**
Codominant	C/C	519 (69.4%)	1054 (75.2%)	1.00	0.011	2775.4	2792.4
	C/T	207 (27.7%)	322 (23%)	**0.77 (0.62-0.94)**			
	T/T	22 (2.9%)	26 (1.8%)	0.58 (0.33-1.04)			
Dominant	C/C	519 (69.4%)	1054 (75.2%)	1.00	0.0041	2774.2	2785.5
	C/T–T/T	229 (30.6%)	348 (24.8%)	**0.75 (0.61-0.91)**			
Recessive	C/C–C/T	726 (97.1%)	1376 (98.2%)	1.00	0.11	2779.9	2791.2
	T/T	22 (2.9%)	26 (1.8%)	0.62 (0.35-1.11)			
Overdominant	C/C-T/T	541 (72.3%)	1080 (77%)	1.00	0.016	2776.7	2788
	C/T	207 (27.7%)	322 (23%)	**0.78 (0.64-0.95)**			
**BDNF rs10835210 association with response Diagnosis (n=2094, crude analysis)**							
**Model**	**Genotype**	**Control**	**Schizophrenia spectrum disorders**	**OR (95% CI)**	**P-value**	**AIC**	**BIC**
Codominant	C/C	197 (27.6%)	342 (24.8%)	1.00	0.35	2689.9	2706.9
	C/A	346 (48.5%)	688 (49.8%)	1.15 (0.92-1.42)			
	A/A	170 (23.8%)	351 (25.4%)	1.19 (0.92-1.53)			
Dominant	C/C	197 (27.6%)	342 (24.8%)	1.00	0.16	2688	2699.3
	C/A-A/A	516 (72.4%)	1039 (75.2%)	1.16 (0.95-1.42)			
Recessive	C/C-C/A	543 (76.2%)	1030 (74.6%)	1.00	0.43	2689.4	2700.7
	A/A	170 (23.8%)	351 (25.4%)	1.09 (0.88-1.34)			
Overdominant	C/C-A/A	367 (51.5%)	693 (50.2%)	1.00	0.58	2689.7	2701
	C/A	346 (48.5%)	688 (49.8%)	1.05 (0.88-1.26)			
**HTR2A rs6313 association with response Diagnosis (n=2053, crude analysis)**							
**Model**	**Genotype**	**Control**	**Schizophrenia spectrum disorders**	**OR (95% CI)**	**P-value**	**AIC**	**BIC**
Codominant	G/G	289 (38.8%)	574 (43.9%)	1.00	0.053	2689.8	2706.7
	G/A	345 (46.3%)	572 (43.7%)	0.83 (0.69-1.01)			
	A/A	111 (14.9%)	162 (12.4%)	**0.73 (0.56-0.97)**			
Dominant	G/G	289 (38.8%)	574 (43.9%)	1.00	0.024	2688.6	2699.9
	G/A-A/A	456 (61.2%)	734 (56.1%)	**0.81 (0.67-0.97)**			
Recessive	G/G-G/A	634 (85.1%)	1146 (87.6%)	1.00	0.11	2691.1	2702.4
	A/A	111 (14.9%)	162 (12.4%)	0.81 (0.62-1.05)			
Overdominant	G/G-A/A	400 (53.7%)	736 (56.3%)	1.00	0.26	2692.4	2703.7
	G/A	345 (46.3%)	572 (43.7%)	0.90 (0.75-1.08)			
**DRD4 rs1800955 association with response Diagnosis (n=2068, crude analysis)**							
**Model**	**Genotype**	**Control**	**Schizophrenia spectrum disorders**	**OR (95% CI)**	**P-value**	**AIC**	**BIC**
Codominant	T/T	238 (34%)	381 (27.8%)	1.00	0.0059	2641.5	2658.4
	T/C	328 (46.9%)	668 (48.8%)	**1.27 (1.03-1.57)**			
	C/C	133 (19%)	320 (23.4%)	**1.50 (1.16-1.95)**			
Dominant	T/T	238 (34%)	381 (27.8%)	1.00	0.0037	2641.4	2652.7
	T/C-C/C	461 (66%)	988 (72.2%)	**1.34 (1.10-1.63)**			
Recessive	T/T-T/C	566 (81%)	1049 (76.6%)	1.00	0.023	2644.6	2655.9
	C/C	133 (19%)	320 (23.4%)	**1.30 (1.03-1.63)**			
Overdominant	T/T-C/C	371 (53.1%)	701 (51.2%)	1.00	0.42	2649.2	2660.4
	T/C	328 (46.9%)	668 (48.8%)	1.08 (0.90-1.29)			

AIC - Akaike information criterion; BIC - Bayesian information criterion; OR - Odds ratio; P-value < 0.05 was considered as significance;

Table S4: Analysis of the association of allele frequency with the study groups (Mood disorders **to** Schizophrenia spectrum disorders).

**BDNF rs6265 association with response Diagnosis (n=1618, crude analysis)**							
**Model**	**Genotype**	**Mood Disorders**	**Schizophrenia spectrum disorders**	**OR (95% CI)**	**P-value**	**AIC**	**BIC**
Codominant	C/C	159 (73.6%)	1054 (75.2%)	1.00	0.66	1276.9	1293
	C/T	51 (23.6%)	322 (23%)	0.95 (0.68-1.34)			
	T/T	6 (2.8%)	26 (1.8%)	0.65 (0.26-1.61)			
Dominant	C/C	159 (73.6%)	1054 (75.2%)	1.00	0.62	1275.4	1286.2
	C/T–T/T	57 (26.4%)	348 (24.8%)	0.92 (0.66-1.28)			
Recessive	C/C–C/T	210 (97.2%)	1376 (98.2%)	1.00	0.39	1274.9	1285.7
	T/T	6 (2.8%)	26 (1.8%)	0.66 (0.27-1.63)			
Overdominant	C/C-T/T	165 (76.4%)	1080 (77%)	1.00	0.83	1275.6	1286.4
	C/T	51 (23.6%)	322 (23%)	0.96 (0.69-1.35)			
**BDNF rs10835210 association with response Diagnosis (n=1598, crude analysis)**							
**Model**	**Genotype**	**Mood Disorders**	**Schizophrenia spectrum disorders**	**OR (95% CI)**	**P-value**	**AIC**	**BIC**
Codominant	A/A	67 (30.9%)	351 (25.4%)	1.00	0.24	1272.8	1288.9
	C/A	101 (46.5%)	688 (49.8%)	1.30 (0.93-1.82)			
	C/C	49 (22.6%)	342 (24.8%)	1.33 (0.90-1.98)			
Dominant	A/A	67 (30.9%)	351 (25.4%)	1.00	0.094	1270.8	1281.6
	C/A-C/C	150 (69.1%)	1030 (74.6%)	1.31 (0.96-1.79)			
Recessive	A/A-C/A	168 (77.4%)	1039 (75.2%)	1.00	0.48	1273.1	1283.9
	C/C	49 (22.6%)	342 (24.8%)	1.13 (0.80-1.59)			
Overdominant	A/A-C/C	116 (53.5%)	693 (50.2%)	1.00	0.37	1272.8	1283.6
	C/A	101 (46.5%)	688 (49.8%)	1.14 (0.86-1.52)			
**HTR2A rs6313 association with response Diagnosis (n=1512, crude analysis)**							
**Model**	**Genotype**	**Mood Disorders**	**Schizophrenia spectrum disorders**	**OR (95% CI)**	**P-value**	**AIC**	**BIC**
Codominant	G/G	78 (38.2%)	574 (43.9%)	1.00	0.12	1198.1	1214.1
	G/A	105 (51.5%)	572 (43.7%)	0.74 (0.54-1.01)			
	A/A	21 (10.3%)	162 (12.4%)	1.05 (0.63-1.75)			
Dominant	G/G	78 (38.2%)	574 (43.9%)	1.00	0.13	1198.1	1208.7
	G/A-A/A	126 (61.8%)	734 (56.1%)	0.79 (0.58-1.07)			
Recessive	G/G-G/A	183 (89.7%)	1146 (87.6%)	1.00	0.39	1199.6	1210.3
	A/A	21 (10.3%)	162 (12.4%)	1.23 (0.76-1.99)			
Overdominant	G/G-A/A	99 (48.5%)	736 (56.3%)	1.00	0.039	1196.1	1206.8
	G/A	105 (51.5%)	572 (43.7%)	**0.73 (0.55-0.98)**			
**DRD4 rs1800955 association with response Diagnosis (n=1579, crude analysis)**							
**Model**	**Genotype**	**Mood Disorders**	**Schizophrenia spectrum disorders**	**OR (95% CI)**	**P-value**	**AIC**	**BIC**
Codominant	T/T	51 (24.3%)	381 (27.8%)	1.00	0.27	1241.5	1257.6
	T/C	115 (54.8%)	668 (48.8%)	0.78 (0.55-1.11)			
	C/C	44 (20.9%)	320 (23.4%)	0.97 (0.63-1.50)			
Dominant	T/T	51 (24.3%)	381 (27.8%)	1.00	0.28	1240.9	1251.6
	T/C-C/C	159 (75.7%)	988 (72.2%)	0.83 (0.59-1.16)			
Recessive	T/T-T/C	166 (79%)	1049 (76.6%)	1.00	0.43	1241.5	1252.2
	C/C	44 (20.9%)	320 (23.4%)	1.15 (0.81-1.64)			
Overdominant	T/T-C/C	95 (45.2%)	701 (51.2%)	1.00	0.11	1239.5	1250.2
	T/C	115 (54.8%)	668 (48.8%)	0.79 (0.59-1.05)			

AIC - Akaike information criterion; BIC - Bayesian information criterion; OR - Odds ratio; P-value < 0.05 was considered as significance;

Table S5: Analysis of the association of allele frequency with the symptom cluster - hallucinations (symptoms observed / no symptoms observed).

**rs6268 association with response Hallucinations (n=1766, crude analysis)**							
**Model**	**Genotype**	**No**	**Yes**	**OR (95% CI)**	**P-value**	**AIC**	**BIC**
Codominant	C/C	430 (73%)	804 (75.1%)	1.00	0.39	2163.4	2179.6
	C/T	150 (25.5%)	245 (22.9%)	0.87 (0.69-1.10)			
	T/T	9 (1.5%)	22 (2%)	1.31 (0.60-2.86)			
Dominant	C/C	430 (73%)	804 (75.1%)	1.00	0.36	2162.4	2173.2
	C/T–T/T	159 (27%)	267 (24.9%)	0.90 (0.71-1.13)			
Recessive	C/C–C/T	580 (98.5%)	1049 (98%)	1.00	0.44	2162.7	2173.5
	T/T	9 (1.5%)	22 (2%)	1.35 (0.62-2.95)			
Overdominant	C/C-T/T	439 (74.5%)	826 (77.1%)	1.00	0.24	2161.9	2172.7
	C/T	150 (25.5%)	245 (22.9%)	0.87 (0.69-1.10)			
**rs10835210 association with response Hallucinations (n=1746, crude analysis)**							
**Model**	**Genotype**	**No**	**Yes**	**OR (95% CI)**	**P-value**	**AIC**	**BIC**
Codominant	A/A	158 (27.1%)	256 (24.2%)	1.00	0.22	2138.7	2154.9
	C/A	294 (50.3%)	525 (49.7%)	1.10 (0.86-1.41)			
	C/C	132 (22.6%)	275 (26%)	1.29 (0.96-1.71)			
Dominant	A/A	158 (27.1%)	256 (24.2%)	1.00	0.21	2138.2	2149
	C/A-C/C	426 (73%)	800 (75.8%)	1.16 (0.92-1.46)			
Recessive	A/A-C/A	452 (77.4%)	781 (74%)	1.00	0.12	2137.3	2148.1
	C/C	132 (22.6%)	275 (26%)	1.21 (0.95-1.53)			
Overdominant	A/A-C/C	290 (49.7%)	531 (50.3%)	1.00	0.81	2139.7	2150.5
	C/A	294 (50.3%)	525 (49.7%)	0.98 (0.80-1.19)			
**rs6313 association with response Hallucinations (n=1651, crude analysis)**							
**Model**	**Genotype**	**No**	**Yes**	**OR (95% CI)**	**P-value**	**AIC**	**BIC**
Codominant	G/G	236 (42.6%)	434 (43.5%)	1.00	0.82	2028.3	2044.4
	G/A	253 (45.7%)	440 (44.1%)	0.95 (0.76-1.18)			
	A/A	65 (11.7%)	124 (12.4%)	1.04 (0.74-1.46)			
Dominant	G/G	236 (42.6%)	434 (43.5%)	1.00	0.74	2026.6	2037.3
	G/A-A/A	318 (57.4%)	564 (56.5%)	0.96 (0.78-1.19)			
Recessive	G/G-G/A	489 (88.3%)	874 (87.6%)	1.00	0.69	2026.6	2037.3
	A/A	65 (11.7%)	124 (12.4%)	1.07 (0.78-1.47)			
Overdominant	G/G-A/A	301 (54.3%)	558 (55.9%)	1.00	0.55	2026.4	2037.1
	G/A	253 (45.7%)	440 (44.1%)	0.94 (0.76-1.16)			
**rs1800955 association with response Hallucinations (n=1729, crude analysis)**							
**Model**	**Genotype**	**No**	**Yes**	**OR (95% CI)**	**P-value**	**AIC**	**BIC**
Codominant	T/T	164 (28.2%)	280 (26.7%)	1.00	0.082	2127.3	2143.5
	T/C	303 (52.1%)	512 (48.8%)	0.99 (0.78-1.26)			
	C/C	115 (19.8%)	258 (24.6%)	1.31 (0.98-1.76)			
Dominant	T/T	164 (28.2%)	280 (26.7%)	1.00	0.51	2129.9	2140.7
	T/C-C/C	418 (71.8%)	770 (73.3%)	1.08 (0.86-1.35)			
Recessive	T/T-T/C	467 (80.2%)	792 (75.4%)	1.00	0.025	2125.3	2136.1
	C/C	115 (19.8%)	258 (24.6%)	**1.32 (1.03-1.69)**			
Overdominant	T/T-C/C	279 (47.9%)	538 (51.2%)	1.00	0.2	2128.7	2139.5
	T/C	303 (52.1%)	512 (48.8%)	0.88 (0.72-1.07)			

AIC - Akaike information criterion; BIC - Bayesian information criterion; OR - Odds ratio; P-value < 0.05 was considered as significance; Yes - symptoms observed; No - no symptoms observed;

Table S6: Analysis of the association of allele frequency with the symptom cluster - deulsions of control (symptoms observed / no symptoms observed).

rs6268 association with response Deulsions of control (n=1766, crude analysis)							
**Model**	**Genotype**	**No**	**Yes**	**OR (95% CI)**	**P-value**	**AIC**	**BIC**
Codominant	C/C	771 (74.8%)	308 (72.5%)	1.00	0.55	1762.5	1778.4
	C/T	242 (23.5%)	111 (26.1%)	1.15 (0.88-1.49)			
	T/T	17 (1.6%)	6 (1.4%)	0.88 (0.35-2.26)			
Dominant	C/C	771 (74.8%)	308 (72.5%)	1.00	0.35	1760.8	1771.4
	C/T–T/T	259 (25.1%)	117 (27.5%)	1.13 (0.88-1.46)			
Recessive	C/C–C/T	1013 (98.3%)	419 (98.6%)	1.00	0.74	1761.6	1772.1
	T/T	17 (1.6%)	6 (1.4%)	0.85 (0.33-2.18)			
Overdominant	C/C-T/T	788 (76.5%)	314 (73.9%)	1.00	0.29	1760.6	1771.1
	C/T	242 (23.5%)	111 (26.1%)	1.15 (0.89-1.49)			
**rs10835210 association with response Deulsions of control (n=1746, crude analysis)**							
**Model**	**Genotype**	**No**	**Yes**	**OR (95% CI)**	**P-value**	**AIC**	**BIC**
Codominant	A/A	274 (26.9%)	94 (22.4%)	1.00	0.12	1738.8	1754.6
	C/A	503 (49.4%)	210 (50%)	1.22 (0.92-1.62)			
	C/C	241 (23.7%)	116 (27.6%)	**1.40 (1.02-1.94)**			
Dominant	A/A	274 (26.9%)	94 (22.4%)	1.00	0.071	1737.8	1748.4
	C/A-C/C	744 (73.1%)	326 (77.6%)	1.28 (0.98-1.67)			
Recessive	A/A-C/A	777 (76.3%)	304 (72.4%)	1.00	0.12	1738.6	1749.2
	C/C	241 (23.7%)	116 (27.6%)	1.23 (0.95-1.59)			
Overdominant	A/A-C/C	515 (50.6%)	210 (50%)	1.00	0.84	1741.1	1751.6
	C/A	503 (49.4%)	210 (50%)	1.02 (0.82-1.29)			
**rs6313 association with response Deulsions of control (n=1651, crude analysis)**							
**Model**	**Genotype**	**No**	**Yes**	**OR (95% CI)**	**P-value**	**AIC**	**BIC**
Codominant	G/G	417 (43.3%)	172 (43.1%)	1.00	0.082	1648.4	1664
	G/A	446 (46.3%)	169 (42.4%)	0.92 (0.71-1.18)			
	A/A	100 (10.4%)	58 (14.5%)	1.41 (0.97-2.03)			
Dominant	G/G	417 (43.3%)	172 (43.1%)	1.00	0.95	1651.4	1661.8
	G/A-A/A	546 (56.7%)	227 (56.9%)	1.01 (0.80-1.28)			
Recessive	G/G-G/A	863 (89.6%)	341 (85.5%)	1.00	0.033	1646.8	1657.3
	A/A	100 (10.4%)	58 (14.5%)	**1.47 (1.04-2.08)**			
Overdominant	G/G-A/A	517 (53.7%)	230 (57.6%)	1.00	0.18	1649.6	1660
	G/A	446 (46.3%)	169 (42.4%)	0.85 (0.67-1.08)			
**rs1800955 association with response Deulsions of control (n=1729, crude analysis)**							
**Model**	**Genotype**	**No**	**Yes**	**OR (95% CI)**	**P-value**	**AIC**	**BIC**
Codominant	T/T	296 (29.3%)	99 (23.4%)	1.00	0.031	1738.6	1754.4
	T/C	497 (49.2%)	212 (50.1%)	1.28 (0.97-1.69)			
	C/C	218 (21.6%)	112 (26.5%)	**1.54 (1.11-2.12)**			
Dominant	T/T	296 (29.3%)	99 (23.4%)	1.00	0.022	1738.3	1748.9
	T/C-C/C	715 (70.7%)	324 (76.6%)	**1.35 (1.04-1.76)**			
Recessive	T/T-T/C	793 (78.4%)	311 (73.5%)	1.00	0.046	1739.6	1750.1
	C/C	218 (21.6%)	112 (26.5%)	**1.31 (1.01-1.70)**			
Overdominant	T/T-C/C	514 (50.8%)	211 (49.9%)	1.00	0.74	1743.5	1754
	T/C	497 (49.2%)	212 (50.1%)	1.04 (0.83-1.30)			

AIC - Akaike information criterion; BIC - Bayesian information criterion; OR - Odds ratio; P-value < 0.05 was considered as significance; Yes - symptoms observed; No - no symptoms observed;

Table S7: Analysis of the association of allele frequency with the symptom cluster - delusions (symptoms observed / no symptoms observed).

**rs6268 association with response Delusions (n=1766, crude analysis)**							
**Model**	**Genotype**	**No**	**Yes**	**OR (95% CI)**	**P-value**	**AIC**	**BIC**
Codominant	C/C	343 (75.4%)	845 (74.2%)	1.00	0.8	1912.1	1928.2
	C/T	105 (23.1%)	272 (23.9%)	1.05 (0.81-1.36)			
	T/T	7 (1.5%)	22 (1.9%)	1.28 (0.54-3.01)			
Dominant	C/C	343 (75.4%)	845 (74.2%)	1.00	0.62	1910.3	1921
	C/T–T/T	112 (24.6%)	294 (25.8%)	1.07 (0.83-1.37)			
Recessive	C/C–C/T	448 (98.5%)	1117 (98.1%)	1.00	0.59	1910.2	1921
	T/T	7 (1.5%)	22 (1.9%)	1.26 (0.53-2.97)			
Overdominant	C/C-T/T	350 (76.9%)	867 (76.1%)	1.00	0.73	1910.4	1921.1
	C/T	105 (23.1%)	272 (23.9%)	1.05 (0.81-1.35)			
**rs10835210 association with response Delusions (n=1746, crude analysis)**							
**Model**	**Genotype**	**No**	**Yes**	**OR (95% CI)**	**P-value**	**AIC**	**BIC**
Codominant	A/A	109 (24.2%)	293 (26%)	1.00	0.73	1889.9	1906
	C/A	231 (51.3%)	557 (49.5%)	0.90 (0.69-1.17)			
	C/C	110 (24.4%)	275 (24.4%)	0.93 (0.68-1.27)			
Dominant	A/A	109 (24.2%)	293 (26%)	1.00	0.45	1888	1898.7
	C/A-C/C	341 (75.8%)	832 (74%)	0.91 (0.70-1.17)			
Recessive	A/A-C/A	340 (75.6%)	850 (75.6%)	1.00	1	1888.5	1899.3
	C/C	110 (24.4%)	275 (24.4%)	1.00 (0.78-1.29)			
Overdominant	A/A-C/C	219 (48.7%)	568 (50.5%)	1.00	0.51	1888.1	1898.8
	C/A	231 (51.3%)	557 (49.5%)	0.93 (0.75-1.16)			
**rs6313 association with response Delusions (n=1651, crude analysis)**							
**Model**	**Genotype**	**No**	**Yes**	**OR (95% CI)**	**P-value**	**AIC**	**BIC**
Codominant	G/G	178 (41.9%)	456 (42.8%)	1.00	0.31	1785.2	1801.1
	G/A	202 (47.5%)	470 (44.1%)	0.91 (0.72-1.15)			
	A/A	45 (10.6%)	139 (13.1%)	1.21 (0.83-1.76)			
Dominant	G/G	178 (41.9%)	456 (42.8%)	1.00	0.74	1785.4	1796
	G/A-A/A	247 (58.1%)	609 (57.2%)	0.96 (0.77-1.21)			
Recessive	G/G-G/A	380 (89.4%)	926 (87%)	1.00	0.19	1783.8	1794.4
	A/A	45 (10.6%)	139 (13.1%)	1.27 (0.89-1.81)			
Overdominant	G/G-A/A	223 (52.5%)	595 (55.9%)	1.00	0.23	1784.1	1794.7
	G/A	202 (47.5%)	470 (44.1%)	0.87 (0.70-1.09)			
**rs1800955 association with response Delusions (n=1729, crude analysis)**							
**Model**	**Genotype**	**No**	**Yes**	**OR (95% CI)**	**P-value**	**AIC**	**BIC**
Codominant	T/T	113 (25.4%)	322 (28.8%)	1.00	0.037	1867.4	1883.4
	T/C	243 (54.6%)	531 (47.5%)	0.77 (0.59-1.00)			
	C/C	89 (20%)	266 (23.8%)	1.05 (0.76-1.45)			
Dominant	T/T	113 (25.4%)	322 (28.8%)	1.00	0.18	1870.1	1880.8
	T/C-C/C	332 (74.6%)	797 (71.2%)	0.84 (0.66-1.08)			
Recessive	T/T-T/C	356 (80%)	853 (76.2%)	1.00	0.1	1869.3	1880.1
	C/C	89 (20%)	266 (23.8%)	1.25 (0.95-1.63)			
Overdominant	T/T-C/C	202 (45.4%)	588 (52.5%)	1.00	0.011	1865.4	1876.2
	T/C	243 (54.6%)	531 (47.5%)	**0.75 (0.60-0.94)**			

AIC - Akaike information criterion; BIC - Bayesian information criterion; OR - Odds ratio; P-value < 0.05 was considered as significance; Yes - symptoms observed; No - no symptoms observed;

Table S8: Analysis of the association of allele frequency with the symptom cluster - catatonic symptoms (symptoms observed / no symptoms observed).

rs6268 association with response Catatonic symptoms (n=1766, crude analysis)							
**Model**	**Genotype**	**No**	**Yes**	**OR (95% CI)**	**P-value**	**AIC**	**BIC**
Codominant	C/C	1002 (74.5%)	101 (78.3%)	1.00	0.26	878.1	894
	C/T	320 (23.8%)	24 (18.6%)	0.74 (0.47-1.18)			
	T/T	23 (1.7%)	4 (3.1%)	1.73 (0.59-5.09)			
Dominant	C/C	1002 (74.5%)	101 (78.3%)	1.00	0.34	877.9	888.5
	C/T–T/T	343 (25.5%)	28 (21.7%)	0.81 (0.52-1.25)			
Recessive	C/C–C/T	1322 (98.3%)	125 (96.9%)	1.00	0.3	877.8	888.4
	T/T	23 (1.7%)	4 (3.1%)	1.84 (0.63-5.40)			
Overdominant	C/C-T/T	1025 (76.2%)	105 (81.4%)	1.00	0.17	877	887.6
	C/T	320 (23.8%)	24 (18.6%)	0.73 (0.46-1.16)			
**rs10835210 association with response Catatonic symptoms (n=1746, crude analysis)**							
**Model**	**Genotype**	**No**	**Yes**	**OR (95% CI)**	**P-value**	**AIC**	**BIC**
Codominant	A/A	349 (26.2%)	33 (26.2%)	1.00	0.55	862.4	878.3
	C/A	653 (49.1%)	67 (53.2%)	1.09 (0.70-1.68)			
	C/C	329 (24.7%)	26 (20.6%)	0.84 (0.49-1.43)			
Dominant	A/A	349 (26.2%)	33 (26.2%)	1.00	0.99	861.6	872.2
	C/A-C/C	982 (73.8%)	93 (73.8%)	1.00 (0.66-1.52)			
Recessive	A/A-C/A	1002 (75.3%)	100 (79.4%)	1.00	0.3	860.6	871.1
	C/C	329 (24.7%)	26 (20.6%)	0.79 (0.51-1.24)			
Overdominant	A/A-C/C	678 (50.9%)	59 (46.8%)	1.00	0.38	860.9	871.4
	C/A	653 (49.1%)	67 (53.2%)	1.18 (0.82-1.70)			
**rs6313 association with response Catatonic symptoms (n=1651, crude analysis)**							
**Model**	**Genotype**	**No**	**Yes**	**OR (95% CI)**	**P-value**	**AIC**	**BIC**
Codominant	G/G	543 (43.2%)	49 (39.5%)	1.00	0.44	838.5	854.1
	G/A	568 (45.2%)	56 (45.2%)	1.09 (0.73-1.63)			
	A/A	145 (11.5%)	19 (15.3%)	1.45 (0.83-2.54)			
Dominant	G/G	543 (43.2%)	49 (39.5%)	1.00	0.42	837.4	847.9
	G/A-A/A	713 (56.8%)	75 (60.5%)	1.17 (0.80-1.70)			
Recessive	G/G-G/A	1111 (88.5%)	105 (84.7%)	1.00	0.23	836.6	847.1
	A/A	145 (11.5%)	19 (15.3%)	1.39 (0.83-2.33)			
Overdominant	G/G-A/A	688 (54.8%)	68 (54.8%)	1.00	0.99	838.1	848.5
	G/A	568 (45.2%)	56 (45.2%)	1.00 (0.69-1.45)			
**rs1800955 association with response Catatonic symptoms (n=1729, crude analysis)**							
**Model**	**Genotype**	**No**	**Yes**	**OR (95% CI)**	**P-value**	**AIC**	**BIC**
Codominant	T/T	382 (28.9%)	27 (21.8%)	1.00	0.092	847.8	863.6
	T/C	656 (49.5%)	61 (49.2%)	1.32 (0.82-2.11)			
	C/C	286 (21.6%)	36 (29%)	**1.78 (1.06-3.00)**			
Dominant	T/T	382 (28.9%)	27 (21.8%)	1.00	0.086	847.6	858.2
	T/C-C/C	942 (71.2%)	97 (78.2%)	1.46 (0.94-2.27)			
Recessive	T/T-T/C	1038 (78.4%)	88 (71%)	1.00	0.064	847.1	857.7
	C/C	286 (21.6%)	36 (29%)	1.48 (0.99-2.24)			
Overdominant	T/T-C/C	668 (50.5%)	63 (50.8%)	1.00	0.94	850.6	861.1
	T/C	656 (49.5%)	61 (49.2%)	0.99 (0.68-1.42)			

AIC - Akaike information criterion; BIC - Bayesian information criterion; OR - Odds ratio; P-value < 0.05 was considered as significance; Yes - symptoms observed; No - no symptoms observed;

Table S9: Analysis of the association of allele frequency with the symptom cluster - neurotic, psychopathic symptoms, habit and impulse disorders (symptoms observed / no symptoms observed).

**rs6268 association with response Neurotic, psychopathic symptoms, habit and impulse disorders (n=1766, crude analysis)**							
**Model**	**Genotype**	**No**	**Yes**	**OR (95% CI)**	**P-value**	**AIC**	**BIC**
Codominant	C/C	576 (75.4%)	400 (74.9%)	1.00	0.78	1763.9	1779.5
	C/T	175 (22.9%)	122 (22.9%)	1.00 (0.77-1.31)			
	T/T	13 (1.7%)	12 (2.2%)	1.33 (0.60-2.94)			
Dominant	C/C	576 (75.4%)	400 (74.9%)	1.00	0.84	1762.4	1772.7
	C/T–T/T	188 (24.6%)	134 (25.1%)	1.03 (0.79-1.33)			
Recessive	C/C–C/T	751 (98.3%)	522 (97.8%)	1.00	0.48	1762	1772.3
	T/T	13 (1.7%)	12 (2.2%)	1.33 (0.60-2.93)			
Overdominant	C/C-T/T	589 (77.1%)	412 (77.2%)	1.00	0.98	1762.4	1772.8
	C/T	175 (22.9%)	122 (22.9%)	1.00 (0.77-1.30)			
**rs10835210 association with response Neurotic, psychopathic symptoms, habit and impulse disorders (n=1746, crude analysis)**							
**Model**	**Genotype**	**No**	**Yes**	**OR (95% CI)**	**P-value**	**AIC**	**BIC**
Codominant	A/A	215 (28.7%)	121 (22.7%)	1.00	0.025	1739.3	1754.8
	C/A	360 (48.1%)	262 (49.2%)	1.29 (0.98-1.70)			
	C/C	174 (23.2%)	150 (28.1%)	**1.53 (1.12-2.09)**			
Dominant	A/A	215 (28.7%)	121 (22.7%)	1.00	0.016	1738.8	1749.1
	C/A-C/C	534 (71.3%)	412 (77.3%)	**1.37 (1.06-1.77)**			
Recessive	A/A-C/A	575 (76.8%)	383 (71.9%)	1.00	0.047	1740.7	1751
	C/C	174 (23.2%)	150 (28.1%)	1.29 (1.00-1.67)			
Overdominant	A/A-C/C	389 (51.9%)	271 (50.8%)	1.00	0.7	1744.5	1754.8
	C/A	360 (48.1%)	262 (49.2%)	1.04 (0.84-1.30)			
**rs6313 association with response Neurotic, psychopathic symptoms, habit and impulse disorders (n=1651, crude analysis)**							
**Model**	**Genotype**	**No**	**Yes**	**OR (95% CI)**	**P-value**	**AIC**	**BIC**
Codominant	G/G	310 (43.1%)	222 (44.3%)	1.00	0.52	1657.9	1673.2
	G/A	331 (46%)	216 (43.1%)	0.91 (0.71-1.16)			
	A/A	79 (11%)	63 (12.6%)	1.11 (0.77-1.62)			
Dominant	G/G	310 (43.1%)	222 (44.3%)	1.00	0.66	1657	1667.2
	G/A-A/A	410 (56.9%)	279 (55.7%)	0.95 (0.76-1.20)			
Recessive	G/G-G/A	641 (89%)	438 (87.4%)	1.00	0.39	1656.4	1666.7
	A/A	79 (11%)	63 (12.6%)	1.17 (0.82-1.66)			
Overdominant	G/G-A/A	389 (54%)	285 (56.9%)	1.00	0.32	1656.2	1666.4
	G/A	331 (46%)	216 (43.1%)	0.89 (0.71-1.12)			
**rs1800955 association with response Neurotic, psychopathic symptoms, habit and impulse disorders (n=1729, crude analysis)**							
**Model**	**Genotype**	**No**	**Yes**	**OR (95% CI)**	**P-value**	**AIC**	**BIC**
Codominant	T/T	218 (28.9%)	148 (28.2%)	1.00	0.94	1737	1752.5
	T/C	370 (49%)	262 (50%)	1.04 (0.80-1.36)			
	C/C	167 (22.1%)	114 (21.8%)	1.01 (0.73-1.38)			
Dominant	T/T	218 (28.9%)	148 (28.2%)	1.00	0.81	1735.1	1745.4
	T/C-C/C	537 (71.1%)	376 (71.8%)	1.03 (0.81-1.32)			
Recessive	T/T-T/C	588 (77.9%)	410 (78.2%)	1.00	0.88	1735.1	1745.4
	C/C	167 (22.1%)	114 (21.8%)	0.98 (0.75-1.28)			
Overdominant	T/T-C/C	385 (51%)	262 (50%)	1.00	0.73	1735	1745.3
	T/C	370 (49%)	262 (50%)	1.04 (0.83-1.30)			

AIC - Akaike information criterion; BIC - Bayesian information criterion; OR - Odds ratio; P-value < 0.05 was considered as significance; Yes - symptoms observed; No - no symptoms observed;

Table S10: Analysis of the association of allele frequency with the symptom cluster - suicidal and auto-aggressive behavior (symptoms observed / no symptoms observed).

**rs6268 association with response Suicidal and auto-aggressive behavior (n=1766, crude analysis)**							
**Model**	**Genotype**	**No**	**Yes**	**OR (95% CI)**	**P-value**	**AIC**	**BIC**
Codominant	C/C	833 (76.1%)	199 (72.9%)	1.00	0.04	1367	1382.7
	C/T	246 (22.5%)	63 (23.1%)	1.07 (0.78-1.47)			
	T/T	16 (1.5%)	11 (4%)	**2.88 (1.32-6.30)**			
Dominant	C/C	833 (76.1%)	199 (72.9%)	1.00	0.28	1370.3	1380.7
	C/T–T/T	262 (23.9%)	74 (27.1%)	1.18 (0.88-1.60)			
Recessive	C/C–C/T	1079 (98.5%)	262 (96%)	1.00	0.013	1365.2	1375.6
	T/T	16 (1.5%)	11 (4%)	**2.83 (1.30-6.17)**			
Overdominant	C/C-T/T	849 (77.5%)	210 (76.9%)	1.00	0.83	1371.4	1381.8
	C/T	246 (22.5%)	63 (23.1%)	1.04 (0.76-1.42)			
**rs10835210 association with response Suicidal and auto-aggressive behavior (n=1746, crude analysis)**							
**Model**	**Genotype**	**No**	**Yes**	**OR (95% CI)**	**P-value**	**AIC**	**BIC**
Codominant	A/A	288 (26.7%)	74 (27.2%)	1.00	0.94	1363.4	1379
	C/A	519 (48.1%)	132 (48.5%)	0.99 (0.72-1.36)			
	C/C	273 (25.3%)	66 (24.3%)	0.94 (0.65-1.36)			
Dominant	A/A	288 (26.7%)	74 (27.2%)	1.00	0.86	1361.5	1371.9
	C/A-C/C	792 (73.3%)	198 (72.8%)	0.97 (0.72-1.31)			
Recessive	A/A-C/A	807 (74.7%)	206 (75.7%)	1.00	0.73	1361.4	1371.8
	C/C	273 (25.3%)	66 (24.3%)	0.95 (0.70-1.29)			
Overdominant	A/A-C/C	561 (51.9%)	140 (51.5%)	1.00	0.89	1361.5	1371.9
	C/A	519 (48.1%)	132 (48.5%)	1.02 (0.78-1.33)			
**rs6313 association with response Suicidal and auto-aggressive behavior (n=1651, crude analysis)**							
**Model**	**Genotype**	**No**	**Yes**	**OR (95% CI)**	**P-value**	**AIC**	**BIC**
Codominant	G/G	447 (43.8%)	108 (40.9%)	1.00	0.55	1310	1325.5
	G/A	460 (45%)	121 (45.8%)	1.09 (0.81-1.46)			
	A/A	114 (11.2%)	35 (13.3%)	1.27 (0.82-1.96)			
Dominant	G/G	447 (43.8%)	108 (40.9%)	1.00	0.4	1308.5	1318.8
	G/A-A/A	574 (56.2%)	156 (59.1%)	1.12 (0.85-1.48)			
Recessive	G/G-G/A	907 (88.8%)	229 (86.7%)	1.00	0.35	1308.3	1318.7
	A/A	114 (11.2%)	35 (13.3%)	1.22 (0.81-1.82)			
Overdominant	G/G-A/A	561 (55%)	143 (54.2%)	1.00	0.82	1309.2	1319.5
	G/A	460 (45%)	121 (45.8%)	1.03 (0.79-1.35)			
**rs1800955 association with response Suicidal and auto-aggressive behavior (n=1729, crude analysis)**							
**Model**	**Genotype**	**No**	**Yes**	**OR (95% CI)**	**P-value**	**AIC**	**BIC**
Codominant	T/T	311 (28.9%)	67 (24.5%)	1.00	0.22	1361.5	1377.1
	T/C	534 (49.7%)	137 (50.2%)	1.19 (0.86-1.65)			
	C/C	230 (21.4%)	69 (25.3%)	1.39 (0.96-2.03)			
Dominant	T/T	311 (28.9%)	67 (24.5%)	1.00	0.15	1360.3	1370.8
	T/C-C/C	764 (71.1%)	206 (75.5%)	1.25 (0.92-1.70)			
Recessive	T/T-T/C	845 (78.6%)	204 (74.7%)	1.00	0.17	1360.6	1371
	C/C	230 (21.4%)	69 (25.3%)	1.24 (0.91-1.69)			
Overdominant	T/T-C/C	541 (50.3%)	136 (49.8%)	1.00	0.88	1362.4	1372.8
	T/C	534 (49.7%)	137 (50.2%)	1.02 (0.78-1.33)			

AIC - Akaike information criterion; BIC - Bayesian information criterion; OR - Odds ratio; P-value < 0.05 was considered as significance; Yes - symptoms observed; No - no symptoms observed;

Table S11: Analysis of the association of allele frequency with the symptom cluster - unlawful and hetero-aggressive behavior (symptoms observed / no symptoms observed).

**rs6268 association with response Unlawful and hetero-aggressive behavior (n=1766, crude analysis)**							
**Model**	**Genotype**	**No**	**Yes**	**OR (95% CI)**	**P-value**	**AIC**	**BIC**
Codominant	C/C	817 (75.2%)	195 (75.9%)	1.00	0.34	1315.6	1331.2
	C/T	251 (23.1%)	54 (21%)	0.90 (0.65-1.26)			
	T/T	19 (1.8%)	8 (3.1%)	1.76 (0.76-4.09)			
Dominant	C/C	817 (75.2%)	195 (75.9%)	1.00	0.81	1315.7	1326.1
	C/T–T/T	270 (24.8%)	62 (24.1%)	0.96 (0.70-1.32)			
Recessive	C/C–C/T	1068 (98.2%)	249 (96.9%)	1.00	0.18	1314	1324.4
	T/T	19 (1.8%)	8 (3.1%)	1.81 (0.78-4.17)			
Overdominant	C/C-T/T	836 (76.9%)	203 (79%)	1.00	0.47	1315.2	1325.6
	C/T	251 (23.1%)	54 (21%)	0.89 (0.64-1.23)			
**rs10835210 association with response Unlawful and hetero-aggressive behavior (n=1746, crude analysis)**							
**Model**	**Genotype**	**No**	**Yes**	**OR (95% CI)**	**P-value**	**AIC**	**BIC**
Codominant	A/A	278 (26%)	73 (28.2%)	1.00	0.68	1316.6	1332.2
	C/A	523 (48.8%)	119 (46%)	0.87 (0.63-1.20)			
	C/C	270 (25.2%)	67 (25.9%)	0.95 (0.65-1.37)			
Dominant	A/A	278 (26%)	73 (28.2%)	1.00	0.47	1314.9	1325.3
	C/A-C/C	793 (74%)	186 (71.8%)	0.89 (0.66-1.21)			
Recessive	A/A-C/A	801 (74.8%)	192 (74.1%)	1.00	0.83	1315.4	1325.8
	C/C	270 (25.2%)	67 (25.9%)	1.04 (0.76-1.41)			
Overdominant	A/A-C/C	548 (51.2%)	140 (54%)	1.00	0.4	1314.7	1325.1
	C/A	523 (48.8%)	119 (46%)	0.89 (0.68-1.17)			
**rs6313 association with response Unlawful and hetero-aggressive behavior (n=1651, crude analysis)**							
**Model**	**Genotype**	**No**	**Yes**	**OR (95% CI)**	**P-value**	**AIC**	**BIC**
Codominant	G/G	431 (42.1%)	112 (46.5%)	1.00	0.059	1231.9	1247.4
	G/A	480 (46.9%)	94 (39%)	0.75 (0.56-1.02)			
	A/A	112 (10.9%)	35 (14.5%)	1.20 (0.78-1.85)			
Dominant	G/G	431 (42.1%)	112 (46.5%)	1.00	0.22	1234.1	1244.4
	G/A-A/A	592 (57.9%)	129 (53.5%)	0.84 (0.63-1.11)			
Recessive	G/G-G/A	911 (89%)	206 (85.5%)	1.00	0.13	1233.3	1243.6
	A/A	112 (10.9%)	35 (14.5%)	1.38 (0.92-2.08)			
Overdominant	G/G-A/A	543 (53.1%)	147 (61%)	1.00	0.026	1230.6	1240.9
	G/A	480 (46.9%)	94 (39%)	**0.72 (0.54-0.96)**			
**rs1800955 association with response Unlawful and hetero-aggressive behavior (n=1729, crude analysis)**							
**Model**	**Genotype**	**No**	**Yes**	**OR (95% CI)**	**P-value**	**AIC**	**BIC**
Codominant	T/T	290 (27.1%)	88 (34.2%)	1.00	0.044	1303.7	1319.3
	T/C	533 (49.9%)	123 (47.9%)	0.76 (0.56-1.04)			
	C/C	246 (23%)	46 (17.9%)	**0.62 (0.42-0.91)**			
Dominant	T/T	290 (27.1%)	88 (34.2%)	1.00	0.025	1303	1313.4
	T/C-C/C	779 (72.9%)	169 (65.8%)	**0.71 (0.53-0.96)**			
Recessive	T/T-T/C	823 (77%)	211 (82.1%)	1.00	0.07	1304.7	1315.1
	C/C	246 (23%)	46 (17.9%)	0.73 (0.51-1.03)			
Overdominant	T/T-C/C	536 (50.1%)	134 (52.1%)	1.00	0.56	1307.7	1318.1
	T/C	533 (49.9%)	123 (47.9%)	0.92 (0.70-1.21)			

AIC - Akaike information criterion; BIC - Bayesian information criterion; OR - Odds ratio; P-value < 0.05 was considered as significance; Yes - symptoms observed; No - no symptoms observed;

Table S12: Analysis of the association of allele frequency with the symptom cluster - affective disorders (symptoms observed / no symptoms observed).

**rs6268 association with response Affective disorders (n=1766, crude analysis)**							
**Model**	**Genotype**	**No**	**Yes**	**OR (95% CI)**	**P-value**	**AIC**	**BIC**
Codominant	C/C	675 (76.3%)	464 (72.2%)	1.00	0.16	2082.1	2098.1
	C/T	196 (22.1%)	164 (25.5%)	1.22 (0.96-1.55)			
	T/T	14 (1.6%)	15 (2.3%)	1.56 (0.75-3.26)			
Dominant	C/C	675 (76.3%)	464 (72.2%)	1.00	0.069	2080.5	2091.1
	C/T–T/T	210 (23.7%)	179 (27.8%)	1.24 (0.98-1.56)			
Recessive	C/C–C/T	871 (98.4%)	628 (97.7%)	1.00	0.29	2082.7	2093.3
	T/T	14 (1.6%)	15 (2.3%)	1.49 (0.71-3.10)			
Overdominant	C/C-T/T	689 (77.8%)	479 (74.5%)	1.00	0.13	2081.4	2092.1
	C/T	196 (22.1%)	164 (25.5%)	1.20 (0.95-1.53)			
**rs10835210 association with response Affective disorders (n=1746, crude analysis)**							
**Model**	**Genotype**	**No**	**Yes**	**OR (95% CI)**	**P-value**	**AIC**	**BIC**
Codominant	A/A	208 (23.9%)	173 (27.1%)	1.00	0.37	2059.3	2075.3
	C/A	440 (50.6%)	314 (49.1%)	0.86 (0.67-1.10)			
	C/C	221 (25.4%)	152 (23.8%)	0.83 (0.62-1.10)			
Dominant	A/A	208 (23.9%)	173 (27.1%)	1.00	0.17	2057.4	2068
	C/A-C/C	661 (76.1%)	466 (72.9%)	0.85 (0.67-1.07)			
Recessive	A/A-C/A	648 (74.6%)	487 (76.2%)	1.00	0.46	2058.8	2069.4
	C/C	221 (25.4%)	152 (23.8%)	0.92 (0.72-1.16)			
Overdominant	A/A-C/C	429 (49.4%)	325 (50.9%)	1.00	0.57	2059	2069.6
	C/A	440 (50.6%)	314 (49.1%)	0.94 (0.77-1.16)			
**rs6313 association with response Affective disorders (n=1651, crude analysis)**							
**Model**	**Genotype**	**No**	**Yes**	**OR (95% CI)**	**P-value**	**AIC**	**BIC**
Codominant	G/G	351 (42.8%)	265 (43.2%)	1.00	0.46	1962.7	1978.5
	G/A	365 (44.5%)	284 (46.2%)	1.03 (0.83-1.29)			
	A/A	104 (12.7%)	65 (10.6%)	0.83 (0.58-1.17)			
Dominant	G/G	351 (42.8%)	265 (43.2%)	1.00	0.89	1962.2	1972.8
	G/A-A/A	469 (57.2%)	349 (56.8%)	0.99 (0.80-1.22)			
Recessive	G/G-G/A	716 (87.3%)	549 (89.4%)	1.00	0.22	1960.8	1971.3
	A/A	104 (12.7%)	65 (10.6%)	0.82 (0.59-1.13)			
Overdominant	G/G-A/A	455 (55.5%)	330 (53.8%)	1.00	0.51	1961.8	1972.4
	G/A	365 (44.5%)	284 (46.2%)	1.07 (0.87-1.32)			
**rs1800955 association with response Affective disorders (n=1729, crude analysis)**							
**Model**	**Genotype**	**No**	**Yes**	**OR (95% CI)**	**P-value**	**AIC**	**BIC**
Codominant	T/T	247 (28.5%)	173 (27.3%)	1.00	0.36	2046.8	2062.7
	T/C	413 (47.6%)	324 (51.2%)	1.12 (0.88-1.43)			
	C/C	207 (23.9%)	136 (21.5%)	0.94 (0.70-1.25)			
Dominant	T/T	247 (28.5%)	173 (27.3%)	1.00	0.62	2046.5	2057.2
	T/C-C/C	620 (71.5%)	460 (72.7%)	1.06 (0.84-1.33)			
Recessive	T/T-T/C	660 (76.1%)	497 (78.5%)	1.00	0.28	2045.6	2056.2
	C/C	207 (23.9%)	136 (21.5%)	0.87 (0.68-1.12)			
Overdominant	T/T-C/C	454 (52.4%)	309 (48.8%)	1.00	0.17	2044.9	2055.6
	T/C	413 (47.6%)	324 (51.2%)	1.15 (0.94-1.41)			

AIC - Akaike information criterion; BIC - Bayesian information criterion; OR - Odds ratio; P-value < 0.05 was considered as significance; Yes - symptoms observed; No - no symptoms observed;

Table S13: Analysis of the association of allele frequency with the symptom cluster - negative symptoms (symptoms observed / no symptoms observed).

**rs6268 association with response Negative symptoms (n=1766, crude analysis)**							
**Model**	**Genotype**	**No**	**Yes**	**OR (95% CI)**	**P-value**	**AIC**	**BIC**
Codominant	C/C	336 (73.5%)	873 (74.7%)	1.00	0.88	1936.6	1952.8
	C/T	112 (24.5%)	273 (23.4%)	0.94 (0.73-1.21)			
	T/T	9 (2%)	22 (1.9%)	0.94 (0.43-2.06)			
Dominant	C/C	336 (73.5%)	873 (74.7%)	1.00	0.61	1934.6	1945.4
	C/T–T/T	121 (26.5%)	295 (25.3%)	0.94 (0.73-1.20)			
Recessive	C/C–C/T	448 (98%)	1146 (98.1%)	1.00	0.91	1934.9	1945.6
	T/T	9 (2%)	22 (1.9%)	0.96 (0.44-2.09)			
Overdominant	C/C-T/T	345 (75.5%)	895 (76.6%)	1.00	0.63	1934.6	1945.4
	C/T	112 (24.5%)	273 (23.4%)	0.94 (0.73-1.21)			
**rs10835210 association with response Negative symptoms (n=1746, crude analysis)**							
**Model**	**Genotype**	**No**	**Yes**	**OR (95% CI)**	**P-value**	**AIC**	**BIC**
Codominant	A/A	128 (27.8%)	284 (24.8%)	1.00	0.23	1926.1	1942.2
	C/A	212 (46.1%)	581 (50.7%)	1.24 (0.95-1.60)			
	C/C	120 (26.1%)	280 (24.4%)	1.05 (0.78-1.42)			
Dominant	A/A	128 (27.8%)	284 (24.8%)	1.00	0.21	1925.5	1936.3
	C/A-C/C	332 (72.2%)	861 (75.2%)	1.17 (0.92-1.49)			
Recessive	A/A-C/A	340 (73.9%)	865 (75.5%)	1.00	0.5	1926.6	1937.4
	C/C	120 (26.1%)	280 (24.4%)	0.92 (0.72-1.18)			
Overdominant	A/A-C/C	248 (53.9%)	564 (49.3%)	1.00	0.092	1924.2	1935
	C/A	212 (46.1%)	581 (50.7%)	1.21 (0.97-1.50)			
**rs6313 association with response Negative symptoms (n=1651, crude analysis)**							
**Model**	**Genotype**	**No**	**Yes**	**OR (95% CI)**	**P-value**	**AIC**	**BIC**
Codominant	G/G	175 (40.2%)	473 (43.6%)	1.00	0.39	1823.5	1839.5
	G/A	208 (47.8%)	477 (44%)	0.85 (0.67-1.08)			
	A/A	52 (11.9%)	134 (12.4%)	0.95 (0.66-1.37)			
Dominant	G/G	175 (40.2%)	473 (43.6%)	1.00	0.22	1821.9	1832.5
	G/A-A/A	260 (59.8%)	611 (56.4%)	0.87 (0.69-1.09)			
Recessive	G/G-G/A	383 (88%)	950 (87.6%)	1.00	0.83	1823.3	1834
	A/A	52 (11.9%)	134 (12.4%)	1.04 (0.74-1.46)			
Overdominant	G/G-A/A	227 (52.2%)	607 (56%)	1.00	0.18	1821.6	1832.2
	G/A	208 (47.8%)	477 (44%)	0.86 (0.69-1.07)			
**rs1800955 association with response Negative symptoms (n=1729, crude analysis)**							
**Model**	**Genotype**	**No**	**Yes**	**OR (95% CI)**	**P-value**	**AIC**	**BIC**
Codominant	T/T	134 (29.4%)	309 (27.1%)	1.00	0.44	1913.4	1929.5
	T/C	228 (50%)	565 (49.6%)	1.07 (0.83-1.39)			
	C/C	94 (20.6%)	265 (23.3%)	1.22 (0.90-1.67)			
Dominant	T/T	134 (29.4%)	309 (27.1%)	1.00	0.36	1912.2	1922.9
	T/C-C/C	322 (70.6%)	830 (72.9%)	1.12 (0.88-1.42)			
Recessive	T/T-T/C	362 (79.4%)	874 (76.7%)	1.00	0.25	1911.7	1922.4
	C/C	94 (20.6%)	265 (23.3%)	1.17 (0.90-1.52)			
Overdominant	T/T-C/C	228 (50%)	574 (50.4%)	1.00	0.89	1913	1923.7
	T/C	228 (50%)	565 (49.6%)	0.98 (0.79-1.22)			

AIC - Akaike information criterion; BIC - Bayesian information criterion; OR - Odds ratio; P-value < 0.05 was considered as significance; Yes - symptoms observed; No - no symptoms observed;
